# Prognostic and Predictive Factors for the Curative Treatment of Esophageal and Gastric Cancer in Randomized Controlled Trials: A Systematic Review and Meta-Analysis

**DOI:** 10.3390/cancers11040530

**Published:** 2019-04-12

**Authors:** Tom van den Ende, Emil ter Veer, Rosa M. A. Mali, Mark I. van Berge Henegouwen, Maarten C. C. M. Hulshof, Martijn G. H. van Oijen, Hanneke W. M. van Laarhoven

**Affiliations:** 1 Department of Medical Oncology, Cancer Center Amsterdam, Amsterdam University Medical Centers, (UMC) location AMC, University of Amsterdam, 1105 AZ Amsterdam, The Netherlands; t.vandenende@amc.uva.nl (T.v.d.E.); e.terveer@amc.uva.nl (E.t.V.); rosa.mali@hotmail.com (R.M.A.M.); m.g.vanoijen@amc.uva.nl (M.G.H.v.O.); 2 Department of Surgery, Cancer Center Amsterdam, Amsterdam University Medical Centers, (UMC) location AMC, University of Amsterdam, 1105 AZ Amsterdam, The Netherlands; m.i.vanbergehenegouwen@amc.uva.nl; 3 Department of Radiotherapy, Cancer Center Amsterdam, Amsterdam University Medical Centers (UMC), Location AMC, University of Amsterdam, 1105 AZ Amsterdam, The Netherlands; m.c.hulshof@amc.uva.nl

**Keywords:** prognosis, chemotherapy, adjuvant, neoadjuvant therapy, stomach neoplasms, esophageal neoplasms

## Abstract

Background: An overview of promising prognostic variables and predictive subgroups concerning the curative treatment of esophageal and gastric cancer from randomized controlled trials (RCTs) is lacking. Therefore, we conducted a systematic review and meta-analysis. Methods: PubMed, EMBASE, CENTRAL, and ASCO/ESMO conferences were searched up to March 2019 for RCTs on the curative treatment of esophageal or gastric cancer with data on prognostic and/or predictive factors for overall survival. Prognostic factors were deemed potentially clinically relevant according to the following criteria; (1) statistically significant (*p* < 0.05) in a multivariate analysis, (2) reported in at least 250 patients, and (3) *p* < 0.05, in ≥33% of the total number of patients in RCTs reporting this factor. Predictive factors were potentially clinically-relevant if (1) the *p*-value for interaction between subgroups was <0.20 and (2) the hazard ratio in one of the subgroups was significant (*p* < 0.05). Results: For gastric cancer, 39 RCTs were identified (*n* = 13,530 patients) and, for esophageal cancer, 33 RCTs were identified (*n* = 8618 patients). In total, we identified 23 potentially clinically relevant prognostic factors for gastric cancer and 16 for esophageal cancer. There were 15 potentially clinically relevant predictive factors for gastric cancer and 10 for esophageal cancer. Conclusion: The identified prognostic and predictive factors can be included and analyzed in future RCTs and be of guidance for nomograms. Further validation should be performed in large patient cohorts.

## 1. Introduction

Gastric and esophageal cancer treated with curative intent both have a poor prognosis with five-year survival rate varying between 30% and 40% [1]. Relapse-related death remains a major challenge for curative treatment. There are several strategies for the curative treatment of gastric cancer including perioperative chemotherapy; i.e., the FLOT regimen (5-fluorouracil, oxaliplatin, and docetaxel) or the MAGIC regimen (epirubicin, 5-fluorouracil, and cisplatin) [2,3]; adjuvant chemotherapy, i.e., S-1 or capecitabine with oxaliplatin [4,5]; and adjuvant chemoradiotherapy, i.e., Intergroup-0116 regimen (5-fluorouracil with radiotherapy) [6,7]. Perioperative chemotherapy is the preferred strategy in Europe, adjuvant chemotherapy in Asia and, in the United States, adjuvant chemo(radio)therapy with or without neoadjuvant treatment is preferred. For the curative treatment of esophageal cancer neoadjuvant chemoradiotherapy, i.e., the European CROSS regimen (carboplatin or paclitaxel with radiotherapy) or the American CALGB 9781 regimen (5-fluorouracil or cisplatin with radiotherapy), [8,9] or neoadjuvant chemotherapy (5-fluorouracil with cisplatin) [10,11] are commonly used strategies. Clinical practice varies between countries, among others due to differences in tumor characteristics and local preferences.

Prognostic and predictive factors are essential in advancing patient tailored medicine. Several clinical and tumor characteristics may identify patients with a poor prognosis, irrespective of the received treatment. For example, patients with lower T and N stage have a higher life expectancy than patients with high T and N stage. Prognostic factors can be identified from Cox proportional hazards, or logistic regression analyses performed within randomized controlled trials (RCTs) or cohort studies. Prognostic factors can be used to stratify patients in RCTs between treatment arms, can serve as baseline factors of interest, and can be included in nomograms.

Predictive factors indicate patient subgroups which could benefit from a specific treatment over the other [12]. For example, HER-2 positive advanced esophagogastric cancer patients benefit from anti HER-2 targeted therapy (trastuzumab), while HER-2 negative patients experience no benefit [13]. Predictive factors can be used in future RCTs to assess the benefit of a certain treatment over the other for a specific subgroup.

There are several prognostic nomograms for the curative treatment of gastric and/or esophageal cancer [14]; for example, the Memorial Sloan Kettering Cancer Center (MSKCC) model predicts survival after a R0 resection for gastric cancer without adjuvant therapy [15]. Most existing data on prognostic factors is based on treatment with surgery alone. In recent years multimodality treatment has become the standard of care for gastric and esophageal cancer. Therefore, it would be interesting to investigate the value of prognostic factors across different treatment settings and compare them to the existing data from large cohort studies or nomograms, as such an overview is lacking. Reviews on prognostic and/or predictive factors in a multimodality setting have retrieved valuable information. For example, in metastasized colorectal and metastasized lung cancer systematic reviews on prognostic factors have found several factors of interest including: performance status (<2), primary tumor resection, smoking history and health related quality of life [16,17].

The current evidence for prognostic and predictive factors for the curative treatment of esophageal and gastric cancer in randomized controlled trials (RCTs) has not yet been systematically reviewed. The aim of this review is to identify potentially clinically relevant prognostic and predictive factors from RCTs to guide future research and clinical care.

## 2. Results

**Figure 1:**
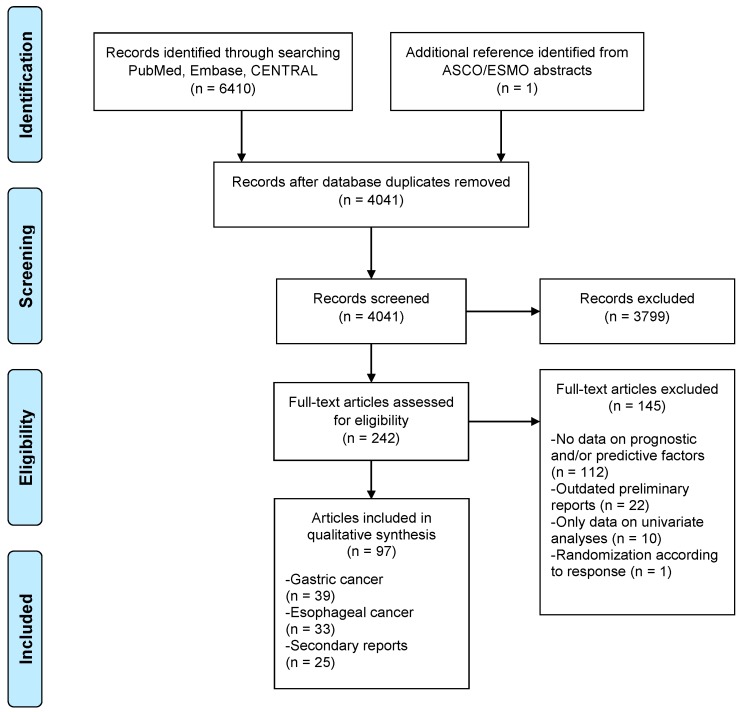
PRISMA flowchart of included studies. ASCO = American Society of Clinical Oncology; CENTRAL = Cochrane Central Register of Controlled Trials; ESMO = European Society for Medical Oncology; PRISMA = Preferred Reporting Items for Systematic Reviews and Meta-Analyses.

In total, 4041 unique references were identified from the PubMed, Embase and CENTRAL databases. Three-thousand-seven-hundred-and-ninety-nine articles were excluded; reasons for exclusion were nonrandomized studies or did not investigate therapy with curative intent for patients without metastases. After title/abstract screening 242 references remained and 145 references were excluded after full text assessment (Figure 1). After searching the ASCO and ESMO conference meeting abstracts one additional reference was identified [18]. Finally, 97 references were identified on 72 original RCTs. In total, 39 studies reported data on prognostic and/or predictive factors for gastric cancer [3,4,5,6,19,20,21,22,23,24,25,26,27,28,29,30,31,32,33,34,35,36,37,38,39,40,41,42,43,44,45,46,47,48,49,50,51,52,53] and 33 studies on esophageal cancer [8,10,11,54,55,56,57,58,59,60,61,62,63,64,65,66,67,68,69,70,71,72,73,74,75,76,77,78,79,80,81,82,83]. In total, there were 25 secondary reports of original RCTs with data on prognostic and/or predictive factors [18,84,85,86,87,88,89,90,91,92,93,94,95,96,97,98,99,100,101,102,103,104,105,106,107]. A full overview of included studies, including baseline characteristics, can be found in Supplementary Tables S1 and S2.

### 2.1. Risk of Bias

The original RCTs were rated according to the Cochrane risk of bias tool (Supplementary Figure S1A,B). For gastric cancer, 19 RCTs (48%) were rated as low risk of bias. Nine studies (23%) were rated as unclear risk of bias on one item. Eight studies (21%) on two items and three (8%) on three or more items. For esophageal cancer, 16 RCTs (48%) were rated as low risk of bias. Three studies (9%) were rated as unclear risk of bias on one item. Six studies (18%) on two items and eight (24%) on three or more items. There were no studies with high risk of bias on one or more domains.

### 2.2. Prognostic Factors

**Figure 2:**
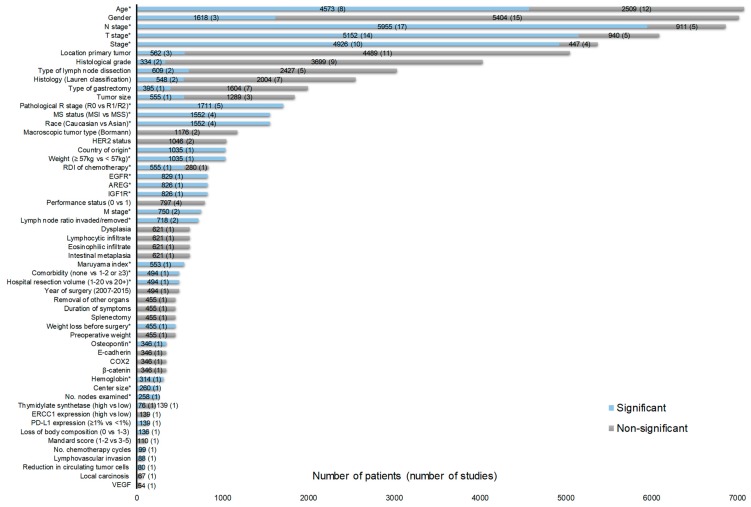
Prognostic factors for the curative treatment of gastric cancer identified from overall survival (OS) multivariate analyses in at least one randomized control trial (RCT). In total, there were 32 RCTs contributing to Figure 2 with 27 investigating adjuvant therapy [4,5,6,19,20,21,22,23,24,25,26,27,29,30,31,32,34,35,36,37,39,40,41,42,43,45,49,52,53] and five investigating neoadjuvant therapy [3,28,33,38,51]. The Mandard score was only assessed in patients who received preoperative chemotherapy according to the MAGIC regimen [93]. * Potentially clinically relevant factors according to the criteria described in the method section. Abbreviations: AREG = Amphiregulin; COX2 = Cyclooxygenase-2; EGFR = Epidermal growth factor receptor; ERCC1 = Excision Repair 1; HER2 = Human epidermal growth factor receptor 2; IGF1R = insulin-like growth factor-1; MS = Microsatellite stability; MSI = Microsatellite instable; MSS = Microsatellite-stable; No.= Number; RDI= Relative dose intensity; PD-L1 = Programmed death-ligand 1; VEGF = Vascular endothelial growth factor.

**Figure 3:**
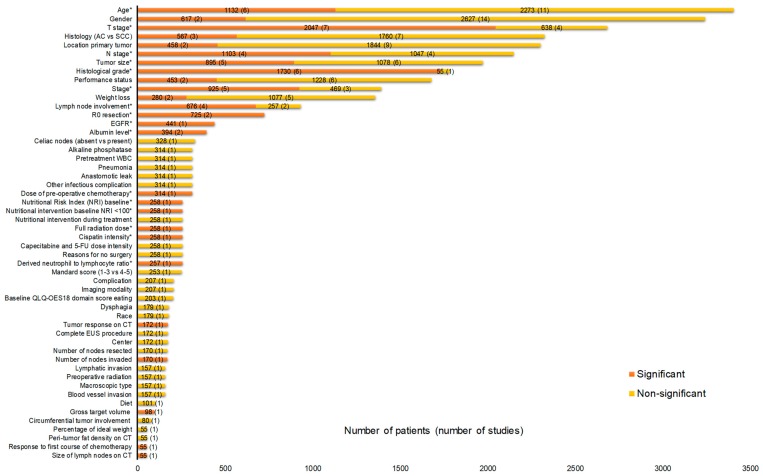
Prognostic factors for the curative treatment of esophageal cancer identified from OS multivariate analyses in at least one RCT. In total, there were 28 RCTs contributing to Figure 3 with 16 investigating neoadjuvant therapy [10,11,54,58,61,62,64,65,66,69,70,73,74,76,80,83], seven definitive chemoradiotherapy [57,60,71,72,75,82,98], three adjuvant therapy [67,77,79], and two radiotherapy alone [63,68]. * Potentially clinically relevant factors according to the criteria described in the method section. AC = Adenocarcinoma; CT = Computed tomography; EGFR = Epidermal growth factor receptor; EUS = Endoscopic ultrasound; NRI = Nutritional risk index; QLQ = Quality of life questionnaire; SCC = squamous cell carcinoma; WBC = White blood cell count.

In total, 55 and 52 factors were identified for gastric and esophageal cancer, respectively (Figure 2 and Figure 3). According to our criteria described in the method section, 23 factors were potentially clinically relevant for gastric cancer and 16 for esophageal cancer (Table 1 and Table 2). In total, for 34 RCTs, one or more HRs were available for factors listed in Figure 2 and Figure 3. The HRs are shown in Supplementary Tables S3 and S4.

### 2.3. Predictive Factors

Subgroup analyses for treatment comparisons in RCTs were reported for 31 predictive factors for gastric cancer and 18 for esophageal cancer. Fifteen potentially clinically relevant predictive factors were identified for gastric cancer (Table 3). For esophageal cancer ten factors were identified (Table 4). A full overview of predictive factors for OS is given in Supplementary Tables S5 and S6.

## 3. Discussion

In total we identified 23 potentially clinically relevant prognostic and 15 predictive factors for the curative treatment of gastric cancer. For esophageal cancer we found 16 prognostic and 10 predictive factors respectively.

### 3.1. Prognostic Factors for Overall Survival in Gastric and Esophageal Cancer

There are several prognostic indices for gastric cancer. The MSKCC nomogram for survival after R0 resection and a model predicting survival after D2 gastrectomy both included age, gender, primary tumor site, tumor size, Lauren histological tumor type (only included in the MSKCC model), number of positive lymph nodes resected, number of negative lymph nodes resected, and depth of invasion [15,109]. The MSKCC model did not improve upon adding weight loss, performance status, hemoglobin, and albumin one year after resection [110]. Other models included lymphovascular invasion, lymph node ratio (invaded/removed), neutrophil to lymphocyte ratio, CRP-to-albumin ratio, and macroscopic type according to the Bormann classification (Bormann gross tumor appearance: type I polypoid, type II fungating, type III ulcerating, and type IV diffuse growing) [111,112,113,114,115]. Several findings are in line with our review including, N stage, age, T stage, and lymph node ratio. However, macroscopic type, gender, primary tumor site, tumor size, and Lauren histological tumor type do not have independent prognostic value based on our data. This could be related to the total number of patients in which each factor was investigated, as a systematic review with over 60,000 patients found that diffuse type tumors had a worse survival compared to intestinal subtype tumors (according to the Lauren classification) [116]. However, we did identify several new factors including microsatellite instability (MSI), AREG, EGFR, IGF1R, osteopontin expression, pretreatment weight, and the Maruyama index (Maruyama index of unresected disease: estimate of the likelihood of nodal involvement for prespecified unresected regional lymph node stations).

For esophageal cancer after esophagectomy, a nomogram based on the Surveillance, Epidemiology, and End Results (SEER) database included age, race, histology, tumor site, tumor size, grade, depth of invasion, number of metastases, and retrieved nodes [117]. Other models included surgical approach, gender, N stage, lymph node ratio, extracapsular lymph node invasion, and the amount of chemotherapy cycles [118,119,120,121]. Our review confirmed the following findings; age, histological grade, T stage, tumor size, N stage, and lymph node involvement. For these factors our review showed relatively consistent results between RCTs with mixed histological subtype populations compared to RCTs with only squamous tumors. Furthermore, we also identified several new factors, such as the derived neutrophil to lymphocyte ratio, EGFR expression, and the baseline nutritional risk index score.

The aforementioned nomograms have several limitations. First, their retrospective nature, which makes them prone to bias, and often absent external validation [14]. Second, each individual nomogram is based on a specific treatment scenario, i.e., after curative resection or during neoadjuvant chemoradiotherapy. A third limitation is the absence of tumor characteristics, i.e., EGFR expression. Our systematic review provides a comprehensive overview of prognostic factors, including tumor characteristics, in RCTs minimizing risk of bias, regardless of treatment strategy neoadjuvant, adjuvant, or after definitive chemoradiotherapy. However, the identified prognostic factors in this review should still be validated in large prospective cohort studies, e.g., the Prospective Observational Cohort study of Oesophageal-gastric cancer Patients (the POCOP project) [122].

### 3.2. Predictive Factors for Overall Survival in Gastric Cancer

We identified several predictive factors, based on patient and tumor characteristics, from the literature. For gastric cancer, intestinal subtype tumors were associated with improved OS to adjuvant chemoradiotherapy compared to diffuse type tumors [6,34]. The CRITICS trial, which investigated the value of adjuvant chemoradiotherapy after neoadjuvant chemotherapy, did not observe a different response between intestinal and diffuse type tumors [123]. Therefore, it seems the subgroup effect is confined to non-pretreated patients after surgery. Potentially, this is related to the differential expression of cancer driver genes in diffuse type gastric cancer [124]. The Cancer Genome Atlas Research Network (TCGA) has also subclassified gastric cancer into four distinct subtypes based on molecular and genomic characterization of 295 primary gastric tumors: Epstein–Barr virus (EBV)-positive tumors, MSI, genomically stable (GS), and chromosomal instable (CIN) tumors [125]. They found that diffuse type histology was often present in the genomically stable subgroup. Hypothetically, these tumors can cope with extensive DNA damage induced by chemoradiotherapy and therefore might not experience as much survival benefit from conventional therapy as chromosomal instable tumors. The overaccumulation of mutations in CIN tumors may thus be exploited by inducing additional DNA damage. Future trials should test the hypothesis if the CIN gastric cancer subtypes experience more survival benefit from chemoradiotherapy than GS (diffuse type tumors).

The MSI gastric cancer subtype seems not to experience survival benefit from conventional chemotherapy. An individual patient data meta-analysis of four RCTs (MAGIC, CLASSIC, ITACA-S, and ARTIST) found, in patients treated with chemotherapy or surgery, that the microsatellite-stable (MS) HR for OS was in favor of chemotherapy, HR = 0.73 (0.61–0.86) [18]. In MSI patients the HR for OS was in favor of surgery alone, HR = 1.49 (0.56–3.96). The *p*-test for interaction was 0.141 in the analysis of the four RCTs, a subanalysis of only the MAGIC and CLASSIC trial revealed a *p* test for interaction of 0.027. The lack of benefit of conventional chemotherapy in MSI patients is also well known in colorectal cancer [126]. The MSI tumors might be candidates for treatment with immunotherapy in the curative setting, as in the metastatic setting gastric and colorectal MSI-high tumors respond well to PD(-L1) inhibitors [127,128]. The same may be true for the EBV+ gastric subtype, as in the CLASSIC trial a combined analysis for disease-free survival (DFS) of a nine-gene-based single patient classifier combined with EBV and MSI status revealed that EBV− tumors experienced DFS benefit from adjuvant chemotherapy while EBV+ tumors did not [129]. In the metastatic setting the EBV gastric subtype was also responsive to PD(-L1) inhibitors [125]. In the future, MSI-high or EBV+ gastric cancer might be (neo)adjuvant treated with a PD(-L1) inhibitor in an RCT to observe potential benefit for these molecular subgroups.

### 3.3. Predictive Factors for Overall Survival in Esophageal Cancer

In our review, we found in one RCT (CROSS trial) that squamous cell cancer (SCC) patients experience more survival benefit from neoadjuvant chemoradiotherapy (nCRT) than adenocarcinoma (AC) patients (*p* interaction: 0.11) [8]. The median survival of SCC was 81.6 months and for AC 43.2 months in the nCRT arm [8]. A pathological complete response (pCR) was observed in 49% of SCC and in 23% of AC patients. A recently conducted RCT (NEOCRTEC5010) in Asian SCC patients observed similar efficacy of nCRT compared to surgery alone. Patients treated with neoadjuvant vinorelbine, cisplatin, and radiotherapy had a median OS of 100.1 months compared to 66.5 months in the surgery alone arm, HR = 0.71 (0.53–0.96). A pCR was found in 43.2% of patients treated with nCRT. The superior efficacy of nCRT in SCC patients compared to AC patients can be related to the molecular basis of both histological subtypes. The TCGA group examined the molecular basis of esophageal cancer [130]. Based on molecular profiling, esophageal SCC tumors resembled head and neck SCC, while esophageal AC resembled the gastric CIN subtype. Chemoradiotherapy is an important modality for patients with SCC of the head and neck and it thus makes sense SCC of the esophagus is also vulnerable to nCRT. Whether neoadjuvant chemotherapy (NAC) is inferior in esophageal SCC compared to nCRT is yet unknown. The NeoRes trial with 181 AC and SCC patients compared NAC to nCRT but found no significant subgroup difference between histological subtypes [83]. Ongoing RCTs, like the Japanese NExT study (JCOG1109), will help elucidate if nCRT is preferred over NAC [131]. For now, SCC is preferably treated with nCRT due to high amount of local responses, while AC can be treated with NAC or nCRT. Future RCTs should focus on identifying clinical or molecular (bio)markers which may help predict response within SCC or AC.

Two nomograms predicting survival benefit in patient subgroups after neoadjuvant chemoradiotherapy found T4 disease, N+, higher grade, and higher T or N status related to survival benefit [119,132]. In our review, we found that in the NEOCRTEC5010 trial cT3 tumors had better OS than cT1-2 and cT4 tumors. For the N0 vs. N+ subgroups in two large RCTs comparing neoadjuvant chemoradiation to surgery alone there was no superior benefit from nCRT in N+ tumors compared to N0 tumors [8,58]. There is, thus, conflicting evidence regarding T and N status and its predictive value. Further validation in large cohorts and RCTs is needed to define T and N subgroups which may experience additional benefit from nCRT. 

### 3.4. Strengths and Limitations

The main strength of this review is the applied method, which enabled us to identify all reported prognostic and predictive factors for the curative treatment of gastric and esophageal cancer from RCTs. We found more prognostic factors than the aforementioned nomograms and several factors were based on more than 1000 randomized patients.

Several limitations should be taken into account when interpreting the results of this review. First, factors from multivariate analyses for prognostic factors including reported hazard ratios were predominantly reported for statistically significant factors (*p* < 0.05). A meta-analysis of hazard ratios could therefore lead to reporting bias where the effect of the HRs will be overestimated.

Second, we devised new criteria for determining clinically relevant prognostic and predictive factors, as there were no available from the literature. Therefore, the results should be read with caution and externally validated as some of the potentially clinically relevant factors are based on one RCT only.

Third, limitations in total sample size could underestimate the independent value of certain prognostic factors. For example, two retrospective analyses based on more than 25,000 patients found a significant survival difference in favor of women for esophageal- and intestinal-type cancer histology to have a better prognosis than diffuse-type tumors for gastric cancer, which we did not find in our review [116,133]. Individual RCTs in our review were mostly based on 200–500 patients, therefore results should be interpreted with caution.

## 4. Materials and Methods

### 4.1. Literature Search

Our systematic review was performed in accordance with the PRISMA (Preferred Reporting Items for Systematic Reviews and Meta-Analyses) guidelines [134]. PubMed, EMBASE, and the Cochrane Central Register of Controlled Trials (CENTRAL) were searched for eligible randomized controlled trials from 1980 up to March 2019. The search strategy consisted of medical subject headings (MeSH) and text words for gastric cancer and esophageal cancer (Supplementary Methods). Moreover, the meeting abstracts from the American Society of Clinical Oncology (ASCO) and European Society for Medical Oncology (ESMO) were searched. The literature search strategy was established and performed by E.t.V. Two authors (T.v.d.E. and RM) screened the titles, abstracts, and full articles independently. Reference lists of studies were cross-checked for potentially missed articles. Disagreements were discussed with a third arbiter (E.t.V. or H.W.M.v.L.) until consensus was reached.

### 4.2. Study Selection and Quality Assessment

Prospective phase II and III randomized controlled trials on the curative treatment of gastric or esophageal cancer were included. Studies which investigated mixed populations of gastric, gastroesophageal junction (GEJ), and esophageal cancer were classified as gastric cancer trials if at least 20% of the total study population included gastric cancer patients (e.g., the MAGIC, CRITICS, ST03, and FFCD-9703 perioperative chemotherapy trials were categorized in the gastric group). Trials with only GEJ patients or a combination of esophageal and GEJ were included in the esophageal group. This is in line with the 8th edition of the American Joint Committee on Cancer classification were GEJ tumors that have their epicenter within the proximal 2 cm of the cardia are regarded as esophageal cancers. Patients could be treated with any combination of chemotherapy (administered either orally, intravenously, or intraperitoneally), targeted agents (e.g., trastuzumab), with surgery, and with radiotherapy. Treatment could be administered either neoadjuvant, adjuvant or perioperative in addition to surgery or without surgery (e.g., definitive chemoradiotherapy). Trials that included patients with distant organ metastases at baseline were excluded. Studies needed to include data on prognostic factors in multivariate regression analyses and/or data from subgroup analyses on predictive variables for overall survival (OS). Quality of the studies was assessed using the Cochrane Risk of Bias tool (version 5.1.0). Items were scored as low, high or unknown risk of bias. Studies with high risk of bias were excluded from the analyses.

### 4.3. Data Extraction and Statistical Analysis

Data extraction for gastric cancer and esophageal cancer was performed by two authors (TvdE, RM). For OS, prognostic factors were identified from the study reports if they were analyzed through multivariable Cox proportional hazards or logistic regression analyses. To determine potentially clinically relevant prognostic factors for OS, we set up new criteria as there were no criteria available from the literature. We considered a prognostic variable potentially clinically relevant when (1) the prognostic factor was statistically significant (*p* < 0.05) in a multivariable or logistic regression analysis in at least one RCT, (2) the combined sample size of RCTs reporting this factor included more than 250 patients, and (3) the combined sample size of RCTs in which this factor was statistically significant (*p* < 0.05) should at least be 33% of the sample size of the RCTs reporting this factor. For example, the variable gender was analyzed in four RCTs (thereby meeting inclusion factor 1) on the curative treatment of gastric cancer with a combined sample size of 1000 patients (and factor 2); however, it was statistically significant in only one RCT with 200 patients (which is only 20% of the total sample size, thus not meeting inclusion factor 3). We assessed gender therefore as not clinically relevant.

Moreover, we extracted from the study reports any given Hazard Ratio (HR) from multivariable analyses with 95% confidence intervals (95% CI) for exploratory reasons only. When the studies used the same comparison to calculate the HR, then these HRs were combined in a random effects pairwise meta-analysis in Review Manger version 5.3, regardless if the factors complied with the aforementioned criteria.

The HRs with 95% CI from predictive factors reported in subgroup analyses were extracted from the study reports. To identify predictive factors which should minimally be included in analyses of future clinical trials (potentially clinically relevant) we set up new criteria as there were none available in the literature. First, the *p*-value for interaction between two or more subgroups should be <0.20. Second, the HR in one of subgroups should be significant (*p* < 0.05). In case no *p*-value for interaction between subgroups was given, we calculated the value in Review Manager version 5.3. The selection strategy for clinically relevant prognostic and predictive factors, as outlined in the method section, can be found in Supplementary Figure S2A,B.

## 5. Conclusions

In this systematic review we identified 39 potentially clinically relevant prognostic and 25 predictive factors from RCTs on the curative treatment of esophageal and gastric cancer. After external validation in large patient cohorts, the identified prognostic factors can be used in day-to-day oncology care and be included in future prognostic models. Moreover, they can serve as a ‘standard’ set to report and to stratify patients between treatments and perform analyses in future trials. The identified predictive factors can be used in future clinical trials to test hypotheses concerning the benefit of treatment in certain subgroups.

## Figures and Tables

**Table 1:** Potentially clinically relevant prognostic factors identified from multivariate OS analyses for the curative treatment of gastric cancer. Highlighted are the subgroups which showed significant (*p* < 0.05) survival benefit over the other subgroup for potentially clinically relevant prognostic factors, if it was possible to identify this from individual studies. Under strategy, the treatment setting of RCTs are listed for each prognostic factor. Metastases (factor: M stage) were discovered after surgery in RCTs.

	Gastric Cancer	
Prognostic Factor	Subgroup	Strategy
Age (years)	**<65** vs. ≥65, **<60** vs. 60–69, **<60** vs. 70–80, **<74** vs. ≥74, Increasing age	Neo [103] Adj [4,5,22,26,31,45,87]
AREG expression	**High** vs. Low	Adj [89]
Center size (No. trial patients)	**Large (≥20)** vs. Small (≤5)	Adj [35]
Comorbidity	**None** vs. 1–2 or ≥3	Neo [103]
Country of origin	**South Korea** vs. China/Taiwan	Adj [5]
EGFR expression	**Negative** vs. Positive	Adj [90]
Hemoglobin	NR	Adj [27]
Hospital resection volume (per year)	**≥20** vs. 1–20	Neo [103]
IGFR1R expression	**Low** vs. High	Adj [89]
Lymph node ratio invaded/removed	**≤0.3** vs. >0.3, **0–25%** vs. >25%	Adj [35,108]
M stage	**M0** vs. M1	Neo, Adj [20,33]
Maruyama index	**<5** vs. ≥5	Adj [86]
Microsatellite instability	**MSI** vs. MSS	Neo and Adj [18]
N stage	**N**− vs. N+, **N0** vs. N1 or N1–2, **pN0** vs. pN1–3, **N0–N1** vs. N2–N3, **N1** vs. N2, **N ≤ 6** vs. N > 6, **N 0–7** vs. N 8–15, **N 0–7** vs. N > 15	Neo [28,33,103], Adj [5,19,20,21,22,24,25,26,27,42,43,84,87]
Number of nodes examined	**>15** vs. ≤15	Adj [26]
Osteopontin expression	**0/1+** vs. 3+	Adj [85]
Pathological R stage	**R0** vs. R1, **R0** vs. R1, R2	Neo [23,28,103], Adj [20,84]
Relative dose intensity (MMC+5-FU+UFT)	**>0.98** vs. <0.98	Adj [87]
Race	Asian vs. Caucasian (benefit in subgroup NR)	Neo and Adj [18]
Stage	**II** vs. IIIA, **II** vs. IIIB, **IB/IIA** vs. IIB/IIIA, **IB/IIA** vs. IIIB, **IB/IIA** vs. IIIC, **IB/II** vs. III/IV	Adj [4,5,20,24,34,37,40,42,45,53]
T stage	**pT0**/**pTis**/**pT1** vs. pT3-4, **pT1** vs. pT2, **pT1** vs. pT3-T4, **pT1-2** vs. pT3-4, **pT3** vs. pT4, **Mucosa** versus Serosa	Neo [33,103], Adj [5,19,21,22,24,25,26,27,32,35,84,87]
Weight	**≥57 kg** vs. <57 kg	Adj [5]
Weight loss (% of normal weight)	Before surgery	Adj [20]

5-FU = Fluorouracil; Adj = Adjuvant; AREG = Amphiregulin; EGFR = Epidermal growth factor receptor; IGF1R = insulin-like growth factor-1; MMC = Mitomycin C; MSI = Microsatellite instable; MSS = Microsatellite-stable; Neo = Neoadjuvant; UFT = Tegafur/uracil; NR = Not reported. Highlighted in bold indicate survival benefit.

**Table 2:** Potentially clinically relevant prognostic factors identified from multivariate OS analyses for the curative treatment of esophageal cancer. Highlighted are the subgroups which showed significant (*p* < 0.05) survival benefit over the other subgroup for potentially clinically relevant prognostic factors, if it was possible to identify this from individual studies. Per factor a subdivision was made between studies which included both adenocarcinoma and squamous cell carcinoma (SCC) and studies which only included squamous cell carcinoma (SCC) patients. Under the heading strategy, the treatment setting of RCTs are listed for each prognostic factor.

Esophageal cancer			
Prognostic factor	Histology	Subgroup	Strategy
Age (years)	AC and SCC	**<60** vs. ≥60, or >70	Neo [65,70]
	AC and SCC	**60–69** vs. <60	dCRT [60]
	SCC	**<70** vs. ≥70	Neo [62]
Albumin level	SCC	**≥4** vs. <4, pretreatment level	Neo, Adj, dCRT [71,107]
Cisplatin intensity	AC and SCC	**≥75%** vs. <75%	dCRT [98]
Derived neutrophil to lymphocyte ratio	AC and SCC	**<2** vs. ≥2	dCRT [98]
Dose of pre-operative chemotherapy	SCC	**≥90%** vs. <90%	Neo [107]
EGFR expression	SCC	**Low** vs. High	Adj [96]
Full radiation dose	AC and SCC	**Yes** vs. No	dCRT [98]
Histological grade	AC and SCC	**Well**/**moderate** vs. Poor	Neo [61,65]
	SCC	**Well** vs. Poor, **Well** vs. Moderate, **Well**/**moderate** vs. Poor	Neo [66,107] Adj [77]
Lymph node involvement	AC and SCC	**No** vs. Yes	Neo [61]
	AC and SCC	**0** vs. ≥1	Neo [58]
	SCC	**No** vs. Yes, **Recurrence 1 node** vs. >1 node	Neo, dCRT [66,75]
N stage	AC and SCC	**cN0** vs. cN1, **pN**− vs. pN+	Neo [64], dCRT [60]
	SCC	**cN0** vs. cN1	Neo [74]
Nutritional Risk Index baseline	AC and SCC	**≥100** vs. <100	dCRT [100]
Nutritional intervention baseline NRI <100	AC and SCC	**Dietary advice** vs. None, **Oral supplements** vs. None, **Major intervention** vs. None	dCRT [100]
R0 resection	AC and SCC	**R0** vs. R1/R2/R3	Neo [76]
	SCC	**R0** vs. R1/R2	Neo [74]
Stage	AC and SCC	**I/II** vs. III, **I/II** vs. III+IV	dCRT [98,101]
	SCC	**I/II** vs. III, **I/II** vs. III+IV, **IIA** vs. IIB	dCRT [71,75], Adj [77]
T stage	AC and SCC	**cT1/T2** vs. cT3 or cT3–4	Neo [83], dCRT [82]
	SCC	**cT1/T2** vs. cT3 or cT4, **pT1–2** vs. pT3	Neo [10,66,80] dCRT [72]
Tumor size (cm)	AC and SCC	**<5** vs. ≥5	Neo [70] dCRT [82]
	SCC	**<3 **vs. ≥3, **<5** vs. ≥5, **<6** vs. ≥6	Neo [62], dRT [63] Adj [77]

AC = Adenocarcinoma; Adj = Adjuvant; dCRT = Definitive chemoradiotherapy; dRT = Definitive radiotherapy; EGFR = Epidermal growth factor receptor; Neo = Neoadjuvant; NRI = Nutritional risk index; SCC = squamous cell carcinoma. Highlighted in bold indicate survival benefit.

**Table 3:** Potentially clinically relevant predictive factors for the curative treatment of gastric cancer. Potentially clinically relevant predictive factors for OS defined as the *p*-value for subgroup interaction between two or more subgroups should be <0.20 and the HR of one of the subgroups was statistically significant (*p* < 0.05). The greater than (>) or smaller than (<) symbols indicate which specific treatment showed a significant survival benefit over the other treatment in a specific patient subgroup highlighted in black. All studies investigated adjuvant therapy after surgery except for Cunningham 2017 and the MAGIC trial.

Gastric Cancer						
Factor	Study	Experimental	vs.	Comparator	HR (95%CI)	*N*
Age (years)						
≥ **70** (vs. <70)	Cunningham 2017 [44]	Peri+Epi+Cis+Cap+BEV	<	Peri+Epi+Cis+Cap	1.67 (1.10–2.52)	1063
Gender						
**Male** (vs. female)	Noh 2014 [5]	Ox+Cap	>	Surg	0.60 (0.45–0.81)	1035
**Female** (vs. male)	Bajetta 2014 [45]	Dtx+IRI+Cis+5-FU/Lv	>	5-FU/Lv	0.73 (0.54–0.98)	1100
**Male** (vs. female)	Smalley 2012 [6]	5-FU/Lv+RT	>	Surg	0.69 (0.55–0.86)	559
T stage						
**T1, T2** (vs. T3, T4)	Noh 2014 [5]	Ox+Cap	>	Surg	0.49 (0.33–0.74)	1035
Nodal stage						
**N0** (vs. N1, N2)	Sasako 2011 [4]	S-1	>	Surg	0.32 (0.13–0.79)	1034
**N1** (vs. N0, N2)	Sasako 2011 [4]	S-1	>	Surg	0.61 (0.44–0.84)	1034
Histopathological grade						
**G1, G2** (vs. G3, G4, GX)	Noh 2014 [5]	Ox+Cap	>	Surg	0.50 (0.31–0.82)	1035
No. of examined lymph nodes						
**15–24** (vs. <15 and ≥25)	Bajetta 2014 [45]	Dtx+IRI+Cis+5-FU/Lv	<	5-FU/Lv	1.48 (1.09–2.01)	1100
Race						
**African American** (vs. other)	Smalley 2012 [6]	5-FU/Lv+RT	>	Surg	0.56 (0.33–0.95)	559
Histology						
**Intestinal** (vs. diffuse)	Smalley 2012 [6]	5-FU/Lv+RT	>	Surg	0.71 (0.54–0.94)	559
Gender and histology						
**Men intestinal** (vs. women intestinal, women diffuse, men diffuse)	Smalley 2012 [6]	5-FU/Lv+RT	>	Surg	0.72 (0.52–0.98)	559
**Woman diffuse** (vs. women intestinal, men intestinal, men diffuse)	Smalley 2012 [6]	5-FU/Lv+RT	<	Surg	2.22 (1.14–4.35)	559
No. nodal metastasis						
**0** vs. (>1)	Sasako 2011 [6]	S-1	>	Surg	0.32 (0.13–0.79)	1034
**1–2** vs. (0 and ≥3)	Sasako 2011 [6]	S-1	>	Surg	0.45 (0.28–0.75)	1034
Stage						
**Stage III** (vs. stage II)	Jeung 2008 [36]	Doxo+5-FU+PAU	>	Doxo+5-FU	0.70 (0.51–0.97)	292
TS expression						
**High** (vs. low)	Sasako 2011 [92]	S-1	>	Surg	0.37 (0.22–0.62)	808
DPD expression						
**High** (vs. low)	Sasako 2011 [92]	S-1	>	Surg	0.52 (0.38–0.72)	807
HER2 expression FISH						
**Non-amplified** (vs. amplified)	Smalley 2012 [94]	5-FU/Lv+RT	>	Surg	0.63 (0.47–0.85)	258
Microsatellite instability						
**MSS** (vs. MSI)	Pietrantonio 2019 [18] (IPD of MAGIC, ARTIST, ITACA-S and CLASSIC)	Perioperative or adjuvant chemotherapy	>	Surg	0.73 (0.61–0.86)	1552
**MSS** (vs. MSI)	Pietrantonio 2019 [18] (IPD of MAGIC and CLASSIC)	Perioperative or adjuvant chemotherapy	>	Surg	0.71 (0.58–0.88)	1552

5-FU = Fluorouracil; BEV = Bevacizumab; Cap = Capecitabine; Cis = Cisplatin; Doxo= Doxorubicin; Dtx = Docetaxel; DPD = Dihydropyrimidine dehydrogenase; Epi = Epirubicin; FISH = Fluorescent in situ hybridization; HER2= Human epidermal growth factor receptor 2; IPD = Individual patient data; IRI = Irinotecan; Lv = Leucovorin; MSI = Microsatellite instable; MSS = Microsatellite-stable; No. = Number; Ox = Oxaliplatin; PAU = polyadenylic–polyuridylic acid; Peri = Perioperative; RT = Radiotherapy; Surg = Surgery only; TS = Thymidylate synthetase.

**Table 4:** Potentially clinically relevant predictive factors for the curative treatment of esophageal cancer. Potentially clinically relevant predictive factors for OS defined as the *p*-value for subgroup interaction between two or more subgroups should be <0.20 and the HR of one of the subgroups was statistically significant (*p* < 0.05). The greater than (>) or smaller than (<) symbols indicate which specific treatment showed a significant survival benefit over the other treatment in a specific patient subgroup highlighted in black. Almost all studies investigated the value of neoadjuvant therapy before surgery except for Crosby 2017 which investigated the value of definitive chemoradiotherapy and Ando 2012 compared neoadjuvant to adjuvant therapy.

Esophageal Cancer						
Factor	Study	Experimental	vs.	Comparator	HR (95%CI)	*N* (histology)
Age (years)						
**>60** (vs. <60)	Boonstra 2011 [54]	Neo+Eto+Cis	>	Surg	0.63 (0.39–1.00)	169 (SCC)
**>70** (vs. <69)	MRC 2002 [11]	Neo+Cis+5-FU	>	Surg	0.64 (0.44–0.91)	802 (AC, SCC)
**<60** (vs. >60)	MRC 2002 [11]	Neo+Cis+5-FU	>	Surg	0.71 (0.55–0.94)	802 (AC, SCC)
**60–69** (<60 and ≥70)	Alderson 2017 [44]	Neo+Epi+Cis+Cap	>	Neo+Cis+5-FU	0.72 (0.57–0.91)	629 (AC)
Gender						
**Male** (vs. female)	Crosby 2017 [98]	dCRT-Cis+Cap+CTX+RT	<	dCRT-Cis+Cap+RT	1.87 (1.26–2.77)	432 (AC, SCC)
**Female** (vs. male)	Stahl 2017 [56]	Neo+Eto+Cis+5-FU/Lv+RT	>	Neo+Cis+5-FU/Lv	0.18 (0.03–0.95)	119 (AC)
**Female** (vs. male)	Liu 2018 [80]	Neo+Vin+Cis+RT	>	Surg	0.34 (0.15–0.80)	451 (SCC)
Histology						
**SCC** (vs. AC)	Shapiro 2015 [8]	Neo+Ptx+Car+RT	>	Surg	0.46 (0.26–0.79)	235 (AC, SCC)
Stage						
**II** (vs. III)	Ando 2012 [10]	Neo+Cis+5-FU	>	Cis+5-FU	0.60 (0.36–0.96)	329 (SCC)
cT stage						
**cT1-2** (vs. cT3)	Ando 2012 [10]	Neo+Cis+5-FU	>	Cis+5-FU	0.36 (0.17–0.80)	330 (SCC)
**cT3** (vs. cT1-2 and cT4)	Liu 2018 [80]	Neo+Vin+Cis+RT	>	Surg	0.56 (0.38–0.82)	451 (SCC)
N stage						
**cN0** (vs. cN1)	Shapiro 2015 [8]	Neo+Ptx+Car+RT	>	Surg	0.49 (0.30–0.80)	231 (AC, SCC)
**N0** (vs. N1)	Alderson 2017 [55]	Neo+Epi+Cis+Cap	>	Neo+Cis+5-FU	0.63 (0.45–0.90)	624 (AC)
Pretreatment weight loss						
**>10%** (vs. 6–10% and <5%)	Boonstra 2011 [54]	Neo+Eto+Cis	>	Surg	0.40 (0.22–0.72)	147 (SCC)
Tumor location						
**Middle** (vs. upper-distal third)	Boonstra 2011 [54]	Neo+Eto+Cis	>	Surg	0.47 (0.29–0.77)	154 (SCC)
**Lower third** (vs. upper/middle and cardia)	MRC 2002 [11]	Neo+Cis+5-FU	>	Surg	0.74 (0.61–0.90)	802 (AC, SCC)
Dysphagia score						
**1** (vs. 0 and >2)	MRC 2002 [11]	Neo+Cis+5-FU	>	Surg	0.66 (0.61–0.85)	754 (AC, SCC)
Reasons for no surgery						
**Comorbidity/poor PS** (vs. patient choice and local extensive disease)	Crosby 2017 [98]	dCRT-Cis+Cap+CTX+RT	<	dCRT-Cis+Cap+RT	3.00 (1.20–7.50)	432 (AC, SCC)

5-FU = Fluorouracil; AC = Adenocarcinoma; Cap = Capecitabine; Car = Carboplatin; Cis = Cisplatin; CTX = Cetuximab; dCRT = Definitive chemoradiotherapy; Epi = Epirubicin; Eto = Etoposide; Lv = Leucovorin; Neo = Neoadjuvant; PS = Performance score; Ptx = Paclitaxel; RT = Radiotherapy; SCC = Squamous cell carcinoma; Surg = Surgery only. These subgroups which show survival benefit are highlighted in bold.

## Supplementary Materials

The following are available online at https://www.mdpi.com/2072-6694/11/4/530/s1, Supplementary Methods, Figure S1A and S1B: risk of bias overview, Figure S2A and S2B: selection process for potentially clinically relevant prognostic and predictive factors, Table S1: Baseline characteristics of the included studies for gastric cancer, Table S2: Baseline characteristics of the included studies for esophageal cancer, Table S3: Multivariate hazard ratios concerning the curative treatment of gastric cancer for OS, Table S4: Multivariate hazard ratios concerning the curative treatment of esophageal cancer for OS, Table S5: Predictive factors for gastric cancer concerning OS, Table S6: Predictive factors for esophageal cancer concerning OS.
