# Supplementary material for: Prognostic and Predictive Factors for the Curative Treatment of Esophageal and Gastric Cancer in Randomized Controlled Trials: A Systematic Review and Meta-Analysis
Source: Cancers (Basel). 2019 Apr 12;11(4):530. doi: 10.3390/cancers11040530 (PMC6521055; doi:10.3390/cancers11040530)
Supplement: Supplementary file 1 [file cancers-11-00530-s001.pdf]

Supplementary Appendix

This appendix has been provided by the authors to give readers additional information about their work.

Supplement to: van den Ende T, ter Veer E, Mali RMA, et al.

Index

Supplementary Methods

Supplementary Figure S1A,B: risk of bias overview

Supplementary Figure S2A,B: selection process for potentially clinically relevant prognostic and predictive factors

Supplementary Table S1 and S2: baseline characteristics of the included studies

Supplementary Table S3 and S4: multivariate hazard ratios for overall survival concerning prognostic factors

Supplementary Table S5 and S6: a full overview of predictive factors for overall survival

Supplementary Methods

Cochrane Central Register of Controlled Trials (CENTRAL)

#	Searches
1	MeSH descriptor: [Esophageal Neoplasms] explode all trees
2	MeSH descriptor: [Stomach Neoplasms] explode all trees
3	(gastric or stomach or esophagus or oesophagus or esophageal or oesophageal) near (neoplasm* or cancer* or carcinoma or adenocarcino* or tumo?r* or malign* neoplasm* neoplasm*):ti,ab,kw (Word variations have been searched)
4	#1 or #2 or #3
5	(chemotherapy or radiotherapy or chemoradiotherapy or radiation or radiochemotherapy) near/1 (adjuvant or neoadjuvant or neo-adjuvant or combined or perioperative or peri-operative or preoperative or pre-operative or postoperative or post-operative):ti,ab,kw (Word variations have been searched)
6	MeSH descriptor: [Gastrectomy] explode all trees
7	MeSH descriptor: [Esophagectomy] explode all trees
8	MeSH descriptor: [Chemotherapy, Adjuvant] explode all trees
9	MeSH descriptor: [Radiotherapy, Adjuvant] explode all trees
10	MeSH descriptor: [Chemoradiotherapy] explode all trees
11	MeSH descriptor: [Radiotherapy] explode all trees
12	#5 or #6 or #7 or #8 or #9 or #10 or #11
13	MeSH descriptor: [Treatment Outcome] explode all trees
14	MeSH descriptor: [Disease-Free Survival] explode all trees
15	MeSH descriptor: [Mortality] explode all trees
16	survival or safe* or mortality or quality of life or QOL:ti,ab,kw (Word variations have been searched)
17	MeSH descriptor: [Quality of Life] explode all trees
18	#13 or #14 or #15 or #16 or #17
19	#4 and #12 and #18 in Trials

EMBASE via Ovid

#	Searches
1	exp *esophagus tumor/ or exp *stomach tumor/ or ((gastric or stomach or esophagus or oesophagus or esophageal or oesophageal) adj (neoplasm* or cancer* or carcinoma or adenocarcino* or tumo?r* or malign* neoplasm* neoplasm*)).ti,ab,kw.
2	gastrectomy/ or *esophagus resection/ or *lymph node dissection/ or exp cancer adjuvant therapy/ or adjuvant chemoradiotherapy/ or cancer radiotherapy/ or gastrectomy.ti,ab,kw. or (exp chemoradiotherapy/ and adjuvant therapy/) or ((chemotherapy or radiotherapy or chemoradiotherapy or radiation or radiochemotherapy) adj1 (adjuvant or neoadjuvant or perioperative or peri-operative or preoperative or pre-operative or postoperative or post-operative)).ti,ab,kw.
3	controlled clinical trial/ or randomized controlled trial/ or "clinical trial (topic)"/ or (randomized or randomised or randomly).ti,ab,kw. or (trial or effecti*).ti.
4	exp treatment outcome/ or exp "quality of life"/ or disease-free survival/ or exp mortality/ or (survival or safe* or mortality or quality of life or QOL).ti,ab,kw.
5	1 and 2 and 3
6	"review"/ not "clinical trial (topic)"/
7	lung.ti.
8	(1 and 2 and 3 and 4) not 6 not 7
9	limit 8 to (dutch or english)

Medline via PubMed

#	Searches
1	esophageal neoplasms/ or stomach neoplasms/ or ((gastric or stomach or esophagus or oesophagus or esophageal or oesophageal) adj (neoplasm* or cancer* or carcinoma or adenocarcino* or tumo?r* or malign* neoplasm* neoplasm*)).ti,ab,kw.
2	exp Gastrectomy/ or Esophagectomy/ or Lymph Node Excision/ or Chemotherapy, Adjuvant/ or Radiotherapy, Adjuvant/ or gastrectomy.ti,ab,kw. or ((exp Chemoradiotherapy/ or exp Radiotherapy/) and (Neoadjuvant Therapy/ or adjuvant or neoadjuvant or neo-adjuvant).ti,ab,kw.) or ((chemotherapy or radiotherapy or chemoradiotherapy or radiation or radiochemotherapy) adj1 (adjuvant or neoadjuvant or neo-adjuvant or combined or perioperative or peri-operative or preoperative or pre-operative or postoperative or post-operative)).ti,ab,kw.
3	exp treatment outcome/ or "Quality of Life"/ or disease-free survival/ or exp Mortality/ or (survival or safe* or mortality or quality of life or QOL).ti,ab,kw.
4	controlled clinical trial/ or randomized controlled trial/ or Clinical Trials as Topic/ or (randomi?ed or randomly).ti,ab,kw. or (trial or effecti*).ti.
5	1 and 2 and 3 and 4
6	"review"/ not Clinical Trials as Topic/
7	lung.ti.
8	(1 and 2 and 3 and 4) not 6 not 7
9	limit 8 to (dutch or english)

Conference search: ***American Society of Clinical Oncology***

Searching journal content for gastric or esophageal (all words) in title or abstract and random* in full text, from earliest publication date through March 2019.

Conference search: ***European Society of Medical Oncology***

Searching journal content for gastric or esophageal (all words) in title or abstract and random* in full text, from earliest publication date through March 2019.

.

Supplementary Figure S1. Risk of bias A= Gastric cancer; B= Esophageal cancer

A

Study	Random sequence generation (selection bias)	Allocation concealment (selection bias)	Blinding of outcome assessment (detection bias)	Incomplete outcome data (attrition bias)	Selective reporting (reporting bias)	Other bias
Allum 1989	+	+	+	+	+	+
Bajetta 2002	+	+	+	+	+	+
Bajetta 2014	+	+	+	+	+	+
Bamias 2010	+	+	+	+	+	+
Basi 2013	+	+	?	+	+	?
Bouche 2005	+	+	+	+	+	+
Chang 2002	+	+	+	+	+	?
Choi 1997	?	?	?	+	+	?
Cunningham 2006	+	+	+	+	+	+
Cunningham 2017	+	+	+	+	+	+
De Vita 2007	+	+	+	+	+	+
Di Costanzo 2008	+	+	+	+	+	+
Feng 2015	+	?	+	+	+	?
Fuchs 2017	+	+	?	+	+	+
Grau 1998	+	?	+	+	+	?
Hallisey 1994	+	+	+	+	+	+
IGTS 1988	+	+	+	+	+	+
Jeung 2008	+	+	?	+	+	?
Kim 1998	+	?	+	+	+	?
Krook 1991	+	?	+	+	+	+
Kuramoto 2009	+	+	+	+	+	+
Lee 2004	+	?	+	+	+	?
Lee 2018	+	+	?	+	+	+
Lise 1995	+	?	+	+	+	+
Ma 2015	+	?	+	+	+	?
Nakajima 1999	+	+	+	+	+	+
Nakajima 2007	+	+	+	+	+	+
Neri 2001	+	?	+	+	+	+
Nio 2004	+	+	+	+	+	?
Noh 2014	+	+	+	+	+	+
Park 2015	+	?	+	+	+	?
Popiela 2004	?	?	?	+	?	?
Sasako 2011	+	+	+	+	+	+
Sautner 1994	?	?	?	+	+	?
Smalley 2012	+	?	+	+	+	+
Verheij 2018	+	+	+	+	+	+
Ychou 2011	+	+	+	+	+	+
Yoshikawa 2016	+	+	+	+	+	+
Zhu 2012	+	?	+	+	+	+

B

Study	Random sequence generation (selection bias)	Allocation concealment (selection bias)	Blinding of outcome assessment (detection bias)	Incomplete outcome data (attrition bias)	Selective reporting (reporting bias)	Other bias
Alderson 2017	+	+	+	+	+	+
Al-Sarraf 1997	+	?	?	+	+	+
Ando 2012	+	+	+	+	+	+
Arnott 1992	+	+	+	+	+	?
Baba 2000	+	+	+	+	+	?
Badwe 1999	+	+	+	+	+	?
Bass 2014	?	?	?	+	+	?
Boonstra 2011	+	+	+	+	+	+
Bosset 1997	+	+	+	+	+	+
Burmeister 2005	+	+	+	+	+	+
Conroy 2014	+	+	+	+	+	+
Crosby 2017	+	+	+	+	+	+
Iizuka 1988	+	?	?	+	+	+
Kelsen 2007	+	?	?	+	+	+
Klevebro 2016	+	+	+	+	+	+
Kumar 2007	+	?	?	+	?	?
Lee 2004	+	+	+	+	?	?
Liu 2018	+	+	+	+	+	+
Ma 2014	?	?	?	+	+	?
Mariette 2014	+	+	+	+	+	+
MRC 2002	+	+	+	+	+	+
Ogoshi 1995	+	?	?	+	+	+
Ruhstaller 2018	+	+	+	+	+	+
Shapiro 2015	+	+	+	+	+	+
Shi 2002	+	?	?	+	+	?
Stahl 2005	+	+	+	+	+	+
Stahl 2017	+	+	+	+	+	+
Suntharalingam 2017	+	+	+	+	+	+
Teoh 2013	+	?	?	+	+	+
Urba 2001	+	?	?	+	+	?
Wang 2016	+	?	?	+	+	?
Xiao 2003	+	?	?	?	?	?
Zhao 2014	+	?	?	+	+	?

Single-centre studies were scored as unknown risk of bias on the item ‘other biases’. The absence of a description of a blinded imaging review committee was not regarded as bias for OS, since the primary outcome OS would not be influenced by this parameter. += Low risk of bias; ?= unknown risk of bias.

Supplementary Figure S2. Selection of potentially clinically relevant prognostic and predictive factors

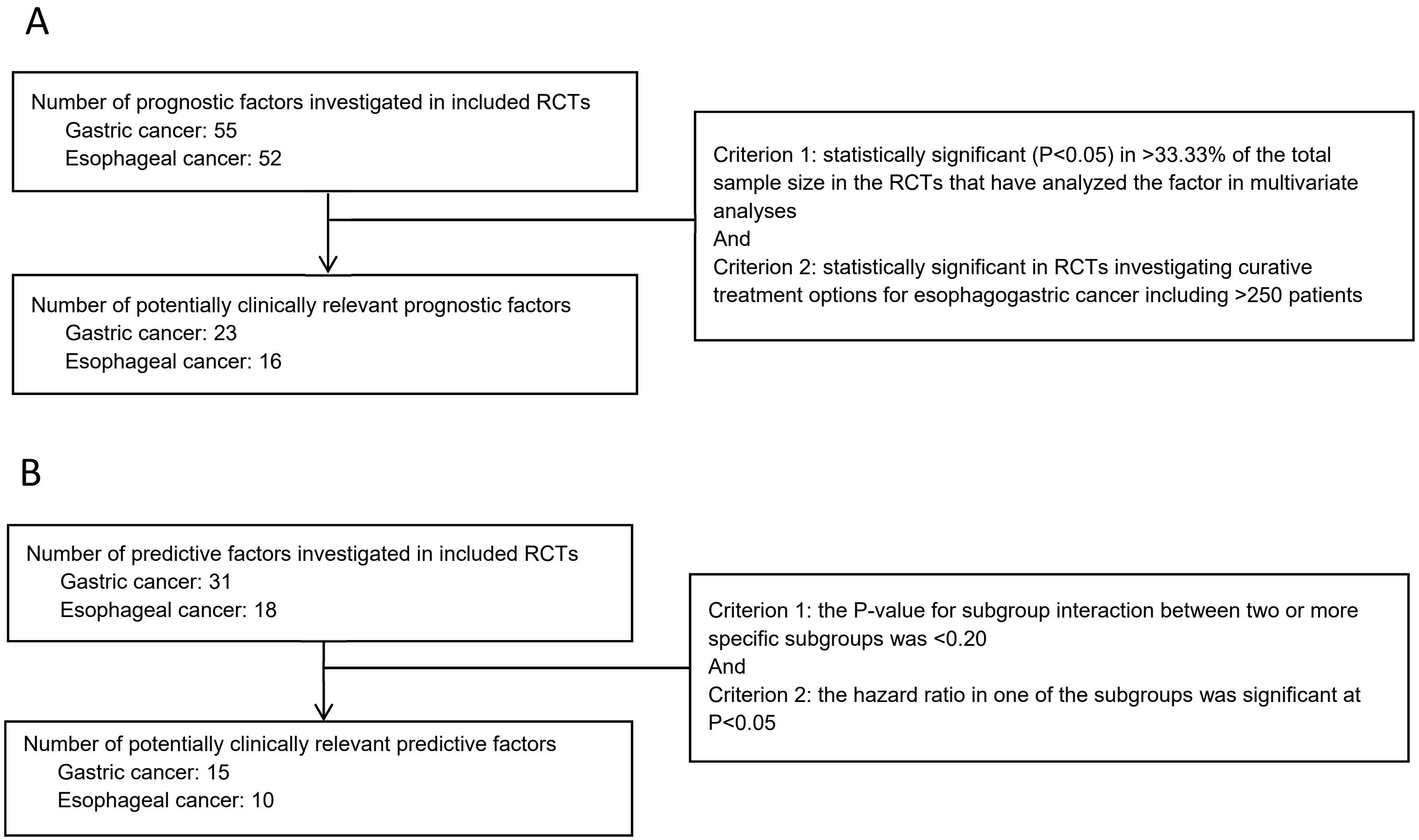

Supplementary Table S1. Baseline characteristics of the included studies for gastric cancer.
In total 39 studies reported data on prognostic and/or predictive factors for gastric cancer.[1-39] In total there were 16 secondary reports of original RCTs with data on prognostic and/or predictive factors.[40-55]

Studies	N	Regimen	Male N (%)	Age, median, (range), y	Stage
IGTS 1988[1]	69	5-FU+MeCCNU+Leva	49 (71)	40 (age 31-60)29>60	T1-4, N- or N+
	75	5-FU+MeCCNU	45 (60)	46 (age 31-60)29>60	
	69	Surg	44 (64)	42 (age 31-60)27>60	
Allum 1989[2]	140	MMC+5-FU+induc (5-FU+Vin+Cycl+MTX)	98 (70)	64	II-IV
	141	MMC+5-FU	97 (68.8)	62	II-IV
	130	Surg	86 (66.2)	63	II-IV
Hallisey 1994[35]	138	Doxo+MMC+5-FU	98 (71)	63	II-IV
	153	RT	99 (65)	65	II-IV
	145	Surg	106 (73)	63	II-IV
Bajetta 2002[3]	135	Doxo+Eto+Cis+5-FU/Lv	81 (59)	57 (23-70)	II-III
	136	Observation	93 (68)	57 (31-70)	II-III
Bajetta 2014[29]	562	Dtx+IRI+Cis+5-FU/Lv	NR	≤ 75 years	II-III
	538	5-FU/Lv	NR	≤ 75 years	II-III
Bamias 2010[4]	72	Dtx+Cis/Car+RT	48 (67)	63 (32-75)	II-III
	71	Dtx+Cis/Car	52 (73)	62 (41-79)	II-III
Basi 2013[6]	28	Peri+Dtx+Cis+5-FU+RT	23 (82.1)	63 (±8)	T1-4, N0-2
	26	5-FU+RT	22 (84.6)	61 (±6)	
Bouche 2005[18]	127	Cis+5-FU	93 (73)	60 (32-82)	II-III
	133	Observation	93 (70)	62 (31-83)	II-III
Chang 2002[7]	131	Doxo+MMC+5-FU	100 (76)	51 (26-70)	I-III
	131	MMC+5-FU	96 (73)	54 (23-74)	I-III
	133	5-FU	99 (74)	53 (21-75)	I-III
Choi 1997[22]	50	Surg+Cis+5-FU+Leva	34 (68)	29 (58) <55 21 (42) > 55	II-IV
	50	Surg+Cis+5-FU	39 (78)	22 (44) <55 28 (56) >55	II-IV
Cunningham 2006[27]	250	Peri+Epi+Cis+5-FU	205 (82)	62 (29-85)	II-III
	253	Surg	191 (76)	62 (23-81)	II-III

Cunningham 2017[28]	530	Peri+Epi+Cis+Cap+BEV	425 (80)	64 (28-82)	II-III
	533	Peri+Epi+Cis+Cap	434 (82)	63 (31-79)	II-III
De Vita 2007[8]	112	Epi+Eto+5-FU/Lv	66 (59)	63 (39-70)	I-III
	113	Observation	65 (58)	62 (41-70)	I-III
Di Costanzo 2008[9]	130	Epi+Cis+5-FU/Lv	79 (61)	59	I-III
	128	Observation	78 (61)	59	I-III
Feng 2015[34]	152	Ox+Cap (Prolonged)	104 (67)	61 (±11)	II-III
	155	Ox+Cap	99 (65)	60 (±10)	II-III
Fuchs 2017[33]	266	Epi+Cis+5-FU+RT	178 (67)	58 (29-83)	I-III
	280	5-FU/Lv+RT	193 (69)	59 (23-81)	I-III
Grau 1998[39]	40	MMC+Tgf	27 (68)	62 (36-75)	I-III
	45	MMC	27 (60)	63 (22-75)	I-III
Jeung 2008[19]	138	Doxo+5-FU+PAU	83 (60)	53 (24-70)	II-III
	142	Doxo+5-FU	90 (63.4)	54 (28-70)	II-III
Kim 1998[23]	49	Doxo+MMC+5-FU+OK432	39 (79.6)	< 60 (37), ≥60 (12)	I-III
	50	Doxo+MMC+5-FU	37 (74)	< 60 (38), ≥60 (12)	I-III
Krook 1991[20]	61	Doxo+5-FU	47 (77)	63 (33-77)	I-III
	64	Observation	51 (80)	62 (38-78)	I-III
Kuramoto 2009[26]	30	Cis (IP+EIPL)+5-FU	13 (43)	63 (±11)*	T3-4, N1-3
	29	Cis (IP)+5-FU	14 (48)	66 (±8)*	
	29	Surg	13 (45)	65 (±7)*	
Lee 2004[24]	32	Epi+Cis+5-FU/Lv	13 (41)	53 (31-61)	III
	29	5-FU	13 (45)	52 (26-66)	III
Lee 2018[36]	75	Dtx+S-1	46 (61)	54 (33-74)	III
	78	Cis+S-1	56 (72)	58 (25-72)	III
Lise 1995[10]	155	Doxo+MMC+5-FU	94 (61)	< 71 years	II-III
	159	Observation	108 (68)	< 71 years	II-III
Park 2015[17]	230	Cis+Cap+RT	143 (62)	56 (28-76)	I-III
	228	Cis+Cap	153 (67)	56 (22-77)	I-III
Popiela 2004[56]	51	Doxo+MMC+5-FU+BCG	34 (66.7)	57 (±12)*	III

	53	Doxo+MMC+5-FU	43 (81.1)	58 (±12)*	III
	52	Surg	31 (59.6)	60 (±11)*	III
Ma 2015[11]	40	Peri+Dtx+Ox+5-FU/Lv+BEV	24 (60)	55*	II-III
	40	Peri+Dtx+Ox+5-FU/Lv	22 (55)	53*	II-III
Smalley 2012[31]	281	5-FU/Lv+RT	202 (72)	60 (25-87)	I-III
	275	Surg	195 (71)	59 (23-80)	I-III
Nakajima 1999[38]	288	MMC+5-FU+UFT	174 (60.4)	40-59 (136, 47.2%) >60 (140, 48.6%)	II-III
	285	Surg	189 (66.3)	40-59 (130, 45.6%) >60 (143, 50.2%)	II-III
Nakajima 2007[12]	93	UFT	75 (70)	63	II-III
	95	Observation	77 (73)	64	II-III
Neri 2001[13]	69	Epi+5-FU/Lv	50 (72.5)	62 (37-73)	II-III
	68	Observation	48 (70.6)	64 (35-74)	II-III
Nio 2004[16]	102	Peri+UFT	70 (69)	64 (±12)	I-IV
	193	UFT	141 (73)	65 (±12)	I-IV
Noh 2014[5]	520	Ox+Cap	373 (72)	56 (±11)*	II-III
	515	Surg	358 (70)	56 (±11)*	II-III
Sasako 2011[30]	529	S-1	367 (69)	63 (27-80)	II-III
	530	Observation	369 (70)	63 (33-80)	II-III
Sautner 1994[15]	33	Cis (IP)	NR	62 (±10)	III-IV
	34	Surg	NR	64 (±11)	III-IV
Verheij 2018[37]	395	Peri+Epi+Cis/Ox+Cap+RT	265 (67)	63 (IQR 56-68)	I-IV
	393	Peri+Epi+Cis/Ox+Cap	264 (67)	62 (IQR 54-69)	I-IV
Ychou 2011[21]	113	Peri+Cis+5-FU	96 (85)	63 (36-75)	I-IV
	111	Surg	91 (82)	63 (38-75)	I-IV
Yoshikawa 2016[32]	20	Neo+Cis+S-1+Surg (4 courses)	12 (60)	63 (47-76)	III-IV
	21	Neo+Cis+S-1+Surg (2 courses)	14 (67)	66 (32-79)	III-IV
	21	Neo+Ptx+Cis+Surg (4 courses)	15 (71)	67 (43-77)	III-IV
	21	Neo+Ptx+Cis+Surg (2 courses)	17 (81)	66 (55-80)	III-IV
Zhu 2012[25]	186	5-FU/Lv+RT	135 (73)	56 (38-73)	I-III

	165	5-FU/Lv	126 (76)	59 (42-75)	I-III

*Mean age. Observation is after curative resection. 5-FU= Fluorouracil; BCG= Bacillus Calmette–Guérin; BEV= Bevacizumab; Cap= Capecitabine; Car= Carboplatin; Cis= Cisplatin; Cycl= Cyclophosphamide; Doxo= Doxorubicin; Dtx= Docetaxel; EIPL= Extensive intraoperative peritoneal lavage; Epi= Epirubicin; Eto= Etoposide; IP= Intraperitoneal; IRI= Irinotecan; Leva= Levamisole; Lv= Leucovorin; MeCCNU= Semustine; MMC= Mitomycin C; MTX= Methotrexate; N= Number; Neo= Neoadjuvant; NR= Not reported; Ox= Oxaliplatin; PAU= polyadenylic–polyuridylic acid; Peri= Perioperative; Ptx= Paclitaxel; RT= Radiotherapy; Surg= Surgery only; Tgfr= Tegafur; UFT= Tegafur/uracil; Vin= Vinblastine; Y= Years

Supplementary Table S2. Baseline characteristics of the included studies for esophageal cancer.
In total there were 33 studies on prognostic and/or predictive factors for esophageal cancer.[57-89] In total there were 9 secondary reports of original RCTs with data on prognostic and/or predictive factors.[90-98]

Studies	N	Regimen	Male N (%)	Age, median, (range), y	Stage	Histology
Alderson 2017[59]	446	Neo+Epi+Cis+Cap	398 (89)	62 (33-80)	II-III	AC (1% SCC)
	451	Neo+Cis+5-FU	412 (91)	62 (27-81)	II-III	
Al sarraf 1997[66]	61	dCRT-Cis+5-FU+RT	49 (80)	65 (44-83)	T1-3, N0-1	AC, SCC
	62	RT alone	49 (79)	63 (44-80)		
	69	dCRT-Cis+5-FU+RT (non-rand)	56 (81)	64 (38-85)		
Ando 2012[62]	164	Neo+Cis+5-FU	144 (88)	61 (34-75)	II-III	SCC
	166	Surg+Cis+5-FU	153 (92)	61 (39-75)	II-III	
Arnott 1992[67]	90	Neo+RT	58 (64)	64 (36-77)*	T1-4, N0, N+, M0/1	AC, SCC
	86	Surg	54 (63)	63 (39-79)*		
Baba 2000[68]	21	Neo+Cis+5-FU/Lv	20 (95)	64 (±7)*	I-IV	SCC
	21	Surg	19 (90)	60 (±8)*	I-IV	
	13	Surg (non-rand)	13 (100)	63 (±8)*	I-IV	
Badwe 1999[69]	43	RT alone	32 (74)	51*	Resectable	SCC
	46	Surg	31 (67)	53*		
Bass 2014[70]	104	Neo+Cis+5-FU	58 (66)	65 (40-75)	I-IV	AC, SCC
	107	Surg	75 (70)	66 (33-75)	I-IV	
Boonstra 2011[57]	85	Neo+Eto+Cis	63 (74)	60 (35-76)	I-III	SCC
	84	Surg	63 (75)	60 (37-79)	I-III	
Bosset 1997[80]	143	Neo+Cis+RT	129 (90)	57 (±8)*	I-II	SCC
	139	Surg	134 (96)	57 (±8)*	I-II	
Burmeister 2005[71]	128	Neo+Cis+5-FU+RT	106 (83)	61 (41-80)	T1-3, N0-1	AC, SCC
	128	Surg	100 (78)	62 (28-83)		
Conroy 2014[63]	134	dCRT-Ox+5-FU/Lv+RT	110 (82)	61 (39-85)	I-IV	AC, SCC
	133	dCRT-Cis+5-FU+RT	107 (80)	60 (41-81)	I-IV	

Crosby 2017[93]	129	dCRT-Cis+Cap+CTX+RT	71 (55)	67 (IQR 45-84)	I-III	AC, SCC
	129	dCRT-Cis+Cap+RT	74 (57)	67 (IQR 36-82)	I-III	
von Dobeln 2018[87]	90	Neo+Cis+5-FU+RT	72 (80)	63 (37-75)	T1-3, N0-N1, M0-M1a	AC, SCC
	91	Neo+Cis+5-FU	77 (85)	63 (38-75)		
Iizuka 1988[72]	104	Peri+RT	95 (91)	(n=44) <59 (n=60) >60	I-III	SCC
	103	Surg+RT	87 (84)	(n=40) <59 (n=63) >60	I-III	
Kelsen 2007[82]	233	Neo+Cis+5-FU	185 (79)	61 (±10)	I-III	AC, SCC
	234	Surg	187 (79)	62 (±10)	I-III	
Kumar 2007[78]	65	dCRT-Cis+RT	43 (66)	58 (24-76)	T1-3, N0-1	SCC
	60	RT alone	49 (82)	56 (34-76)		
Lee 2004[24]	51	Neo+Cis+5-FU+RT	46 (90)	63 (42-73)	II-III	SCC
	50	Surg	47 (94)	63 (39-75)	II-III	
Liu 2018[86]	224	Neo+Vin+Cis+RT	190 (85)	56 (31-70)	IIB-III	SCC
	227	Surg	177 (78)	58 (35-70)		
Ma 2014[81]	49	dCRT-Cis+RT	35 (71)	52 (40-69)	I-IV LRR	SCC
	49	RT alone	32 (65)	54 (38-71)	I-IV LRR	
Mariette 2014[64]	98	Neo+5-FU+Cis+RT	87 (89)	58 (40-76)	I-II	AC, SCC
	97	Surg	80 (83)	58 (37-74)	I-II	
MRC 2002[58]	400	Neo+Cis+5-FU+(RT)	306 (77)	63 (36-84)	Resectable	AC, SCC
	402	Neo+(RT)/Surg alone	297 (74)	62 (30-80)		
Ogoshi 1995[73]	56	Surg+Bleo+Tgf+PSK	48 (86)	59 (41-77)	I-IV	SCC
	38	Surg+RT+PSK	34 (89)	59 (44-81)	I-IV	
	49	Surg+Bleo+Tgf	46 (94)	57 (47-76)	I-IV	
	31	Surg+RT	26 (84)	65 (44-82)	I-IV	
Ruhstaller 2018[88]	149	Peri-CTX+Neo+Dtx+Cis+RT	130 (87)	61 (56-68)	T2-4a, N0-3, M0	AC, SCC
	151	Neo+Dtx+Cis+RT	133 (88)	61 (53-68)		
Shapiro 2015[61]	178	Neo+Ptx+Car+RT	134 (75)	60 (55-67)	II-III	AC, SCC
	188	Surg	152 (81)	60 (53-66)	II-III	
Shi 2002[74]	49	RT (CAHF)	37 (76)	57 (44-73)	I-III	SCC

	52	RT (LCAF)	31 (60)	56 (39-73)	I-III	
Stahl 2005[75]	86	Neo+Eto+Cis+5-FU/Lv+RT	69 (80)	57 (37-70)	II-III	SCC
	86	dCRT+Eto+Cis+5-FU/Lv+RT	69 (80)	57 (36-71)	II-III	
Stahl 2017[60]	60	Neo+Eto+Cis+5-FU/Lv+RT	54 (90)	61	II-III	AC
	59	Neo+Cis+5-FU/Lv	54 (92)	56	II-III	
Suntharalingam 2017[89]	168	dCRT+Ptx+Cis+CTX+RT	130 (82)	65 (39-87)	T1-4, N0-N1, M0-M1a	AC, SCC
	176	dCRT+Ptx+Cis+RT	146 (86)	63 (32-85)		
Teoh 2013[77]	36	dCRT-Cis+5-FU+RT	NR	NR	I-III	SCC
	44	Surg	NR	NR	I-III	
Urba 2001[76]	50	Neo+Vin+Cis+5-FU+RT	42 (84)	62 (39-75)*	Resectable	AC, SCC
	50	Surg	43 (86)	64 (42-75)*		
Wang 2016[83]	106	Surg+RT	85 (80)	(n=34) <50 (n=72) ≥50	II	SCC
	106	Surg	85 (80)	(n=33) <50 (n=73) ≥50	II	
Xiao 2003[85]	220	Surg+RT	188 (86)	(n=157) <59 (n=63) ≥60	I-III	SCC
	275	Surg	201 (73)	(n=189) <59 (n=86) ≥60	I-III	
Zhao 2014[65]	175	Peri+Ptx+Cis+5-FU	152 (87)	59 (26-89)	Resectable	SCC
	171	Neo+Ptx+Cis+5-FU	145 (85)	59 (23-90)		

*Mean age. 5-FU= Fluorouracil; AC= Adenocarcinoma; Bleo= Bleomycin; CAHF= Continuous accelerated hyperfractionated; Cap= Capecitabine; Car= Carboplatin; Cis= Cisplatin; CTX= Cetuximab; dCRT= Definitive chemoradiotherapy; Epi= Epirubicin; Eto= Etoposide; LCAF= Late-course accelerated hyperfractionated; Peri= Perioperative; Lv= Leucovorin; N= Number; Neo= Neoadjuvant; Non rand= Non randomized patients; NR= Not reported; Ox= Oxaliplatin; PSK= Polysaccharide K; Ptx= Paclitaxel; RT= Radiotherapy; SCC= Squamous cell carcinoma; Surg= Surgery only; Tgf= Tegafur; Vin= Vinblastine; Y= Years

Supplementary Table S3. Multivariate hazard ratios concerning the curative treatment of gastric cancer for OS.
Reported HRs were extracted from the study reports. This overview is for exploratory purposes only as the majority of the studies did not report HRs. A random effect pairwise meta-analysis was performed in case there was more than one HR for the same two subgroups available. A HR > 1 indicates a worse survival for subgroup category 1 compared to category 2.
The HRs marked with a superscript number one were based on studies which treated patients neoadjuvant or the meta-analysis included both neoadjuvant and adjuvant studies. The other HRs are based on studies investigating adjuvant treatment.

Gastric cancer					
**Factor**	**Category 1**	**Category 2**	**N**	**Studies**	**HR (95%CI)**
N stage	# metastatic nodes	NR	80	1[11]	3.32 (1.56-7.04)[1]
	pN1	pN0	494	1[53]	1.53 (1.09-2.15)[1]
	pN2	pN0	494	1[53]	3.43 (2.28-5.15)[1]
	pN3	pN0	494	1[53]	8.41 (4.47-15.83)[1]
	N+	N0	1869	3[1,5,40]	2.30 (2.23-2.37)
	N2	N0-N1	143	1[4]	2.92 (1.66-5.15)
	N2-N3	N0-N1	675	3[3,4,9]	2.48 (1.51-4.05)
T stage	Serosa	Mucosa	621	1[40]	1.63 (1.27-2.08)
	pT2	pT0/pTis/pT1	494	1[53]	2.69 (1.49-4.86)[1]
	pT3	pT0/pTis/pT1	494	1[53]	5.35 (2.92-9.80)[1]
	pT4	pT0/pTis/pT1	494	1[53]	6.10 (2.98-12.45)[1]
	pT2	pT1	213	1[1]	2.31 (2.14-2.49)
	pT3-4	pT1	213	1[1]	3.12 (2.93-3.33)
	T3-4	T1-2	1970	5[3-5,9,18]	2.10 (1.77-2.49)
Location primary tumor	Upper third	Lower third	1319	2[1,29]	2.12 (0.59-7.59)
	Upper third	Middle third	1593	3[1,3,29]	1.75 (0.64-4.80)
	Upper third	Other	473	2[1,18]	1.96 (1.12-3.46)
	Multicentric	Upper third	1106	1[29]	1.16 (0.86-1.55)
	Other	GEJ	1035	1[5]	1.96 (0.71-5.26)
	Antrum	Other	1035	1[5]	1.02 (0.78-1.32)
Gender	Male	Female	3461	4[5,29,30,53]	1.10 (0.93-1.29)[1]
Age (years)	Age	NR	143	1[4]	1.03 (1.01-1.06)
	Increasing age		494	1[53]	1.02 (1.01-1.03)[1]
	Increase of 10 years		1364	2[9,29]	1.22 (1.01-1.48)
	≥65	<65	1035	1[5]	1.33 (1.00-1.76)
	60-69	<60	826	1[30]	1.30 (1.10-1.53)
	70-80	<60	826	1[30]	1.69 (1.22-2.35)
Histology (Lauren)	Diffuse	Intestinal	840	2[29,53]	1.47 (1.14-1.90)[1]
	Mixed	Intestinal	494	1[53]	1.27 (0.67-2.40)
Residual tumor	R1	R0	939	2[40,53]	1.98 (1.54-2.54)[1]
	R1, R2	R0	80	1[11]	2.00 (1.13-3.51)[1]
Stage	IIIA	IB+II	2207	3[5,29,30]	1.92 (1.44-2.55)
	IIIB	IB+II	2207	3[5,29,30]	3.31 (2.29-4.78)
	IIIC	IB+IIA	346	1[29]	12.51 (5.42-28.85)
	III+IV (M0)	IB+II	1564	2[17,29]	3.48 (2.64-4.58)
Histological differentiation	G3, G4, GX	G1, G2	2487	4[5,19,29,30]	1.28 (1.09-1.50)
Country	China/Taiwan	South Korea	1035	1[5]	1.63 (1.13-2.35)
Weight	<57 kg	≥57 kg	1035	1[5]	1.37 (1.04-1.82)
LN ratio invaded/removed	>25-30%	≤25-30%	718	2[18,99]	3.22 (2.54-4.09)
E-cadherin expression	Abnormal	Normal	346	1[41]	1.14 (0.79-1.65)
Osteopontin expression	2+	0/1+	346	1[41]	1.40 (0.97-2.04)
	3+	0/1+	346	1[41]	1.64 (1.15-2.34)
B-Catenin expression	Abnormal	Normal	346	1[41]	1.11 (0.76-1.62)
COX-2 expression	Normal	Overexpressed	346	1[41]	1.03 (0.60-1.79)
Type of LND	D1	D2,D3	1106	1[29]	1.16 (0.87-1.56)
# nodes examined	≤15	>15	258	1[9]	2.13 (1.49-3.13)
CTC reduction	NR	NR	80	1[11]	2.48 (1.21-5.08)[1]
Center size	Medium	Large	260	1[18]	1.49 (0.98-2.26)
	Small	Large	260	1[18]	1.79 (1.23-2.59)
Mandard score	3-5	1-2	110	1[49]	1.32 (0.69-2.52)[1]
HER2 status baseline	HER2 negative	HER2 positive	217	1[47]	1.72 (0.94-3.13)[1]
HER2 status resection	HER2 positive	HER2 negative	1046	2[46,47]	1.04 (0.78-1.39)[1]
HER2 BDISH+, IHC (0-1+)	HER2 positive	HER2 negative	217	1[47]	1.17 (0.77-1.77)[1]
AREG	Low	High	826	1[45]	1.52 (1.19-1.96)
IGF1R	High	Low	826	1[45]	1.72 (1.33-2.21)

EGFR expression	Positive	Negative	829	1[46]	1.50 (1.02-2.15)
Maruyama index	≥5	<5	553	1[42]	1.90 (1.30-2.80)
MS status	MSS	MSI	1552	4[52]	2.08 (1.23-3.45)[1]
Hospital volume (resections per year)	Low volume (1-20)	High volume (21+)	494	1[53]	1.45 (1.06-2.00)[1]
PD-L1 status	<1% positive	≥1% positive	139	1[55]	2.63 (1.28-5.56)
ERCC1 expression	High	Low	139	1[55]	1.24 (0.63-2.42)
Loss of body composition	1-3	0	136	1[54]	2.89 (1.66-5.01)
Year of surgery	2007-2015		494	1[53]	1.04 (0.97-1.12)[1]
Comorbidity	1-2	None	494	1[53]	1.61 (1.02-2.56)[1]
	≥3	None	494	1[53]	1.64 (1.00-2.69)[1]
Thymidylate synthase expression	High	Low	139	1[55]	1.58 (0.80-3.07)

AREG= Amphiregulin; BDISH= Brightfield double in situ hybridization; CTC= Circulating tumor cells; GEJ= Gastroesophageal junction; HER2= Human epidermal growth factor receptor 2; EGFR= Epidermal growth factor receptor; IGF1R= insulin-like growth factor-1; IHC= Immunohistochemistry; LN= Lymph node; LND= Lymph node dissection; MS= microsatellite; MSS= microsatellite stable; MSI= Microsatellite instable; No.= Number

Supplementary Table S4. Multivariate hazard ratios concerning the curative treatment of esophageal cancer for OS.
Reported HRs were extracted from the study reports. This overview is for exploratory purposes only as the majority of the studies did not report HRs. A random effect pairwise meta-analysis was performed in case there was more than one HR for the same two subgroups available. A HR > 1 indicates a worse survival for subgroup category 1 compared to category 2. The treatment strategies of the RCTs involved are outlined in the table. Subgroup analyses per factor for squamous cell carcinoma and adenocarcinoma were not performed due to the fact most studies included both subtypes.

Esophageal cancer						
**Factor**	**Category 1**	**Category 2**	**N**	**Studies**	**HR (95%CI)**	**Strategy**
Age	>60	≤60	1095	4	1.15 (0.97-1.36)	Neo[71,72,86,87]
	≥65	<65	572	2	1.32 (1.00-1.75)	Neo[100], dCRT[93]
	>70	≤70	155	2	1.71 (1.17-2.50)	Neo[68,76]
	Continous		542	2	1.87 (0.76-4.63)	Adj[91], dRT[74]
Gender	Male	Female	1575	6	1.43 (1.18-1.73)	Neo[64,67,100], dCRT[86,87,93]
	Gender		207	1	1.01 (0.68-1.48)	Neo[72]
	Gender		101	1	1.56 (0.81-3.02)	dRT[74]
	Gender		441	1	0.75 (0.59-0.97)	Adj[91]
T stage	cT3	cT1-2	1071	4	1.87 (1.35-2.57)	Neo[86,87,100], dCRT[78]
	cT4	cT1-2	451	1	2.71 (1.45-5.05)	Neo[86]
	cT3-T4	cT1-2	328	1	1.55 (1.07-2.26)	dCRT[89]
	T1-T4		441	1	1.25 (1.05-1.50)	Adj[91]
	Invasion adventitia+	No invasion	207	1	1.76 (1.13-2.73)	Neo[72]
N stage	cN1	cN0	946	3	1.08 (0.80-1.44)	Neo[86,87,100]
	N2-N3	N0-N1	441	1	1.25 (1.11-1.41)	Adj[91]
Nodal involvement	Yes	No	879	5	1.76 (1.35-2.30)	Neo[64,67,72,76,81]
Celiac nodes	Absent	Present	328	1	1.05 (0.76-1.47)	dCRT[89]
Recurrence nodes	≥2	<1	98	1	1.02 (1.01-1.03)	dCRT[81]
Size of N nodes on CT	≥1 cm	<1 cm	55	1	1.54 (1.03-2.31)	Neo[68]
Performance score	1	0	854	4	1.01 (0.93-1.08)	Neo[87,100], dCRT[66,93]
	1-2	0	328	1	1.48 (1.14-1.91)	dCRT[89]
	≥1	0	320	2	1.56 (1.12-2.17)	Neo[64], dCRT[78]
Histology	AC	SCC	1972	8	1.04 (0.83-1.30)	Neo[64,70,71,76,82,87], dCRT[89,93]
Stage	Stage I, IIA, III		101	1	3.85 (0.51-28.57)	dRT[74]
	II	I	196	1	1.66 (0.95-2.87)	Neo[64]
	III/IV	I/II	301	2	1.77 (1.28-2.46)	dCRT[81,96]
	III	I/II	258	1	1.52 (1.05-2.21)	dCRT[93]
	IIB	IIA	336	1	1.74 (1.26-2.40)	Adj[83]
Tumor length (cm)	>5	≤5	529	3	1.48 (1.19-1.84)	Neo[76] dCRT[89], dRT[74]
	≥6	<6	55	1	1.49 (1.01-2.21)	Neo[68]

	<4	4-6	258	1	1.02 (0.65-1.61)	dCRT[93]
	6-8	<4	258	1	1.20 (0.72-2.00)	dCRT[93]
	>8	<4	258	1	1.50 (0.91-2.50)	dCRT[93]
	≥7	<7	207	1	1.18 (0.82-1.69)	Neo[72]
	≥3	<3	336	1	1.54 (1.10-1.96)	Adj[83]
Histological differentiation	Poor	Well/moderate	1730	6	1.45 (1.22-1.73)	Neo[71,72,100,101], Adj[83,91]
	Moderate	Well	314	1	2.94 (1.05-8.28)	Neo[100]
	Unknown	Well	314	1	2.42 (0.71-8.28)	Neo[100]
Tumor location	Lower third	Upper third	414	2	1.50 (0.76-2.96)	Neo[76,100]
	Upper third	Middle third	765	2	1.25 (0.82-1.90)	Neo[86,100]
	Middle third	Lower third	176	1	1.92 (0.95-3.89)	Neo[67]
	Proximal/middle	Cardia/distal	181	1	1.39 (0.78-2.45)	Neo[87]
	Lower, upper, middle		207	1	1.14 (0.78-1.65)	Neo[72]
	Lower, upper, middle, cervical		101	1	1.07 (0.68-1.68)	dRT[74]
Weight loss	≥10%	<10%	443	1	1.07 (0.86-1.32)	Neo[82]
Residual tumor	R1	R0	443	1	2.42 (1.71-3.41)	Neo[82]
	R2	R0	443	1	4.18 (3.05-5.72)	Neo[82]
	R3	R0	443	1	4.45 (3.33-5.94)	Neo[82]
Diet	Full diet, semiliquid, liquid, fasting		101	1	1.15 (0.71-1.87)	dRT[74]
Tumor response (Eto+Cis+5-FU/Lv)	No	Yes	172	1	3.33 (2.13-5.26)	Neo[75]
PR to first course of chemo	No	Yes	55	1	2.94 (1.35-6.42)	Neo[68]
Albumin level	<4.0	≥4.0	314	1	2.63 (1.52-4.55)	Neo[100]
Pretreatment WBC	<10000	≥10000	314	1	1.27 (0.43-3.70)	Neo[100]
ALP	≥300	<300	314	1	1.03 (0.50-2.12)	Neo[100]
Pneumonia after operation	Present	Absent	314	1	1.66 (0.87-3.17)	Neo[100]
Anostomotic leak	Present	Absent	314	1	1.04 (0.47-2.26)	Neo[100]
Other infectious complication	Absent	Present	314	1	1.45 (0.65-3.23)	Neo[100]
Dose of preoperative chemo	<90%	≥90%	314	1	2.38 (1.30-4.35)	Neo[100]
Reasons for no surgery	Patient choice	Local extent disease	258	1	1.03 (0.71-1.49)	dCRT[93]
	Patient choice	Comorbidity/poor PS	258	1	1.02 (0.60-1.72)	dCRT[93]
Full radiation dose	No	Yes	258	1	2.06 (1.21-3.49)	dCRT[93]
Cis intensity	<75%	≥75%	258	1	1.80 (1.12-2.89)	dCRT[93]
Cap/5-FU intensity	≥75%	<75%	258	1	1.18 (0.75-1.85)	dCRT[93]
Complication	Present, absent		207	1	1.42 (0.97-2.07)	Neo[72]
X-ray type	Spiral, others		207	1	1.19 (0.84-1.69)	Neo[72]
GTV of RT	≥5cm3	<5cm3	98	1	4.27 (2.18-8.37)	dCRT[81]
No. N nodes invaded	1-3	0	170	1	4.10 (2.20-7.80)	Neo[92]
	>3	0	170	1	4.10 (1.90-8.50)	Neo[92]
EGFR expression	High	Low	441	1	1.45 (1.14-1.86)	Adj[91]
dNLR	≥2	<2	257	1	1.64 (1.17-2.29)	dCRT[94]
Nutritional Risk Index baseline	<100	≥100	258	1	12.45 (5.24-29.57)	dCRT[102]
NI baseline NRI <100	None	Dietary advice	258	1	8.33 (1.96-33.33)	dCRT[102]
	None	Oral supplements	258	1	7.69 (2.56-25)	dCRT[102]

	None	Major intervention	258	1	7.69 (2-33.33)	dCRT[102]
Nutritional intervention	Dietary advice	None	258	1	1.18 (0.59-2.39)	dCRT[102]
	Oral supplements	None	258	1	1.00 (0.54-1.85)	dCRT[102]
	None	Major intervention	258	1	1.89 (0.67-5.26)	dCRT[102]
Baseline QLQ-OES18	Domain score eating		203	1	1.08 (1.00-1.15)	dCRT[96]
Mandard score	1-3	4-5	253	1	1.35 (0.92-1.98)	Neo[98]

5-FU= Fluorouracil; AC= Adenocarcinoma; Adj= Adjuvant; ALP= Alkaline phosphatase; Cap= Capecitabine; Cis= Cisplatin; cm= Centimeters; CT= Computed tomography; dCRT= Definitive chemoradiotherapy; dNLR= Derived neutrophil to lymphocyte ratio; dRT= Definitive radiotherapy; EGFR= Epidermal growth factor receptor; Eto= Etoposide; GTV= Gross target volume; Lv= Leucovorin; Neo= Neoadjuvant; NI= Nutritional intervention; No.= Number; NRI= Nutritional risk index; PR= Partial response; PS= Performance score; QLQ= Quality of life questionnaire; RT= Radiotherapy; SCC= squamous cell carcinoma; WB= White blood cell count

Supplementary Table S5. Predictive factors for gastric cancer concerning OS

Comparison	Studies	N	Factor	Categories	HR	P interaction
Ox+Cap vs Surg	Noh 2014[5]	1035	Country	China/Taiwan	0.66 (0.35-1.27)	0.98
				South Korea	0.67 (0.51-0.88)	
		1035	Stage	II	0.54 (0.34-0.87)	0.55
				IIIA	0.75 (0.52-1.10)	
				IIIB	0.67 (0.39-1.13)	
		1035	Age	< 65 years	0.67 (0.50-0.91)	0.86
				≥ 65 years	0.70 (0.44-1.12)	
		1035	Gender	Female	0.93 (0.57-1.51)	0.14
				Male	0.60 (0.45-0.81)	
		1035	Nodal status	N0	0.79 (0.32-1.95)	0.71
				N1 or N2	0.67 (0.51-0.87)	
		1035	Weight	< 57 kg	0.67 (0.47-0.95)	0.94
				≥ 57 kg	0.68 (0.47-0.99)	
		1035	Primary tumor	T1, T2, T2a, T2b	0.49 (0.33-0.74)	0.03
				T3, T4	0.87 (0.62-1.21)	
		1035	Histopathological grade	G1, G2	0.50 (0.31-0.82)	0.14
				G3, G4, GX	0.77 (0.57-1.04)	
		1035	Primary tumor location	Antrum	0.68 (0.48-0.97)	1.00
				GEJ	0.63 (0.09-4.45)	
				Other	0.69 (0.47-1.00)	
Peri+Epi+Cis+5-FU vs Surg	Cunningham 2006[27]	503	Age	< 60 years	0.67 (0.45-1.02)	0.56
				60-69 years	0.94 (0.60-1.49)	
				≥ 70 years	0.76 (0.43-1.38)	
		503	Gender	Male	0.82 (0.60-1.11)	0.50
				Female	0.68 (0.39-1.24)	
		503	Performance status	0	0.74 (0.54-1.06)	0.63
				1	0.83 (0.53-1.32)	

		503	Primary tumor location	Lower esophagus	0.78 (0.40-1.61)	0.25
				GEJ	0.49 (0.23-1.09	
				Stomach	0.84 (0.62-1.15)	
Peri+Epi+Cis+5-FU vs Surg	Okines 2013[47]	217	HER2 baseline	HER2+	0.74 (0.14-3.77)	0.77
				HER2-	0.58 (0.41-0.82)	
S-1 vs Surg	Sasako 2011[30]	1034	Gender	Male	0.68 (0.52-0.88)	0.82
				Female	0.64 (0.43-0.96)	
		1034	Age	< 60 years	0.55 (0.38-0.80)	0.44
				60-69 years	0.68 (0.47-0.98)	
				70-80 years	0.78 (0.53-1.15)	
		1034	Stage (Japanese)	II	0.51 (0.34-0.77)	0.29
				IIIA	0.71 (0.51-0.98)	
				IIIB	0.79 (0.52-1.21)	
		1034	Stage (UICC 6th)	IIA	0.52 (0.36-0.75)	0.41
				IIIA	0.67 (0.46-0.96)	
				IIIB	0.86 (0.51-1.43)	
				IV	0.78 (0.42-1.46)	
		1034	Tumor stage (UICC 6th)	T2	0.65 (0.47-0.90)	0.78
				T3	0.69 (0.51-0.93)	
				T4	0.41 (0.10-1.73)	
		1034	Nodal stage (Japanese)	N0	0.32 (0.13-0.79)	0.09
				N1	0.61 (0.44-0.84)	
				N2	0.84 (0.61-1.15)	
		1034	No. Of nodal metastasis	0	0.32 (0.13-0.79)	0.21
			(UICC 6th)	1-6	0.61 (0.44-0.83)	
				7-15	0.78 (0.53-1.14)	
				> 16	0.93 (0.48-1.80)	
		1034	No. Of nodal metastasis	0	0.32 (0.13-0.79)	0.09
			(UICC 7th)	1-2	0.45 (0.28-0.75)	
				3-6	0.74 (0.49-1.11)	

				>7	0.82 (0.59-1.14)	
		1031	Histological type	Differentiated	0.67 (0.48-0.94)	0.98
				Undifferentiated	0.67 (0.51-0.90)	
S-1 vs Surg	Sasako 2015[48]	808	TS expression	Low	0.76 (0.56-1.02)	0.02
				High	0.37 (0.22-0.62)	
		807	DPD expression	Low	0.85 (0.56-1.28)	0.07
				High	0.52 (0.38-0.72)	
		807	TP expression	Low	0.78 (0.52-1.17)	0.21
				High	0.56 (0.40-0.77)	
		807	OPRT expression	Low	0.68 (0.50-0.91)	0.38
				High	0.53 (0.33-0.85)	
S-1 vs Surg	Ichikawa 2017[45]	827	AREG expression	Low	0.57 (0.41-0.79)	0.29
				High	0.74 (0.51-1.08)	
		827	IGF1R expression	Low	0.72 (0.49-1.06)	0.31
				High	0.55 (0.40-0.76)	
S-1 vs Surg	Terashima 2012[46]	829	EGFR status	Negative	0.63 (0.48-0.81)	0.67
				Positive	0.75 (0.37-1.49)	
		829	HER2 status	Negative	0.63 (0.49-0.83)	1.00
				Positive	0.64 (0.34-1.20)	
Peri+Cis+5-FU vs Surg	Ychou 2011[21]	224	Primary tumor location	Esophagus only	1.14 (0.47-2.80)	0.26
				GEJ	0.57 (0.39-0.83)	
				Stomach	0.92 (0.42-2.06)	
Peri+Epi+Cis/Ox+Cap+RT vs Peri+Epi+Cis/Ox+Cap	Cats 2018[37]	788	Histology	Intestinal	1.35 (0.85-2.17)	0.24
				Diffuse	0.88 (0.58-1.35)	
				Mixed	1.46 (0.44-4.78)	
				Unknown	0.93 (0.62-1.41)	
		788	Location	GEJ	1.12 (0.61-2.06)	0.16
				Proximal	1.33 (0.79-2.24)	
				Middle	1.17 (0.75-1.81)	

				Distal	0.77 (0.49-1.19)	
		788	Gender	Male	1.30 (0.96-1.76)	0.00
				Female	0.68 (0.44-1.04)	
		788	Age	< 60 years	0.94 (0.64-1.38)	0.07
				60-69 years	1.40 (0.93-2.10)	
				≥ 70 years	0.81 (0.48-1.35)	
Peri+Epi+Cis+Cap+BEV vs Peri+Epi+Cis+Cap	Cunningham 2017[28]	1063	Age	< 60 years	1.06 (0.80-1.42)	0.05
				60 to <70 years	0.91 (0.70-1.19)	
				≥ 70 years	1.67 (1.10-2.52)	
		1063	Gender	Male	1.08 (0.89-1.31)	0.97
				Female	1.07 (0.70-1.65)	
		1063	Performance status	0	1.06 (0.86-1.31)	0.74
				1	1.13 (0.83-1.53)	
		1063	Primary tumor location	Stomach	1.27 (0.94-1.73)	0.44
				GEJ type III	0.86 (0.60-1.24)	
				GEJ type I/II	1.08 (0.79-1.47)	
				Lower esophagus	1.01 (0.61-1.67)	
		592	Gastric tumor stage	I	0.68 (0.29-1.60)	0.63
				II	1.11 (0.73-1.69)	
				IIIa	1.27 (0.89-1.81)	
				IIIb	1.10 (0.55-2.20)	
				IV	0.76 (0.32-1.80)	
		451	Esophageal tumor stage	II	1.35 (0.83-2.19)	0.28
				III	0.98 (0.71-1.36)	
Dtx+IRI+Cis+5-FU/Lv vs 5-FU/Lv	Bajetta 2014[29]	1100	Gender	Male	1.17 (0.94-1.47)	0.01
				Female	0.73 (0.54-0.98)	
		1100	Age	< 60 years	0.86 (0.65-1.13)	0.30
				60-69 years	1.09 (0.82-1.46)	
				> 70 years	1.03 (0.69-1.54)	

		1100	Histology	Diffuse	1.19 (0.88-1.63)	0.16
				Intestinal	0.86 (0.60-1.22)	
		1100	Stage	Ib/II	1.05 (0.70-1.57)	0.39
				IIIa	0.74 (0.53-1.03)	
				IIIb	1.26 (0.85-1.85)	
				IV	1.08 (0.78-1.50)	
		1100	N stage (AJCC6th)	N0	0.61 (0.26-1.45)	0.78
				N1 (1-6)	1.07 (0.78-1.48)	
				N2 (7-15)	0.95 (0.71-1.26)	
				N3 (>15)	1.07 (0.75-1.52)	
		1100	N stage (AJCC7th)	N0	0.61 (0.26-1.45)	0.85
				N1 (1-2)	1.19 (0.65-2.18)	
				N2 (3-6)	0.99 (0.68-1.45)	
				N3a (7-15)	0.95 (0.71-1.26)	
				N3b (>15)	1.07 (0.75-1.52)	
		1100	# examined nodes	<15	0.80 (0.47-1.37)	0.15
				15-24	1.48 (1.09-2.01)	
				≥ 25	0.80 (0.63-1.03)	
		1100	Lymphnode dissection	D1	0.93 (0.66-1.30)	0.67
				D2-D3	1.01 (0.82-1.25)	
		1100	Primary tumor location	Upper third	1.14 (0.70-1.86)	0.36
				Middle third	0.99 (0.69-1.41)	
				Lower third	0.92 (0.66-1.28)	
				Multicentric	0.93 (0.67-1.30)	
Doxo+Eto+Cis+5-FU/Lv vs Surg	Bajetta 2002[3]	274	T stage	T1/2	0.85 (0.46-1.32)	0.59
				T3/4	1.04 (0.69-1.65)	
		274	N stage	N-/N+ ≤6	0.98 (0.64-1.54)	0.67
				N+ >6	0.85 (0.51-1.30)	
		274	Primary tumor location	Middle, distal third	0.89 (0.59-1.31)	0.63
				Upper third, whole	1.04 (0.64-2.26)	

		274	Combination T, N stage and location	T1/2, N-/N+ ≤6, middle, distal third	0.87 (0.44-1.41)	0.98
				T1/2, N-/N+ ≤6, upper third, whole	0.98 (0.54-2.27)	
				T1/2, N+ >6, middle, distal third	0.80 (0.33-1.27)	
				T1/2 N+ >6 upper third, whole	0.91 (0.43-1.78)	
				T3/4, N-/N+ ≤6, middle, distal third	1.03 (0.65-1.84)	0.97
				T3/4, N-/N+ ≤6, upper third, whole	1.08 (0.68-3.39)	
				T3/4, N+ >6, middle, distal third	0.91 (0.55-1.46)	
				T3/4 N+ >6 upper third, whole	1.03 (0.62-2.54)	
Neo+Ptx+Cis vs Neo+Cis+S-1	Yoshikawa 2016[32]	83	Age	≥ 70 years	1.56 (0.58-4.24)	0.28
				< 70 years	0.74 (0.30-1.82)	
		83	Gender	Male	1.64 (0.69-3.91)	0.13
				Female	0.56 (0.19-1.64)	
		83	Macroscopic type	Scirrhous	1.25 (0.50-3.10)	0.66
				Non scirrhous	0.93 (0.37-2.35)	
		83	Esophageal invasion	Positive	1.42 (0.38-5.35)	0.58
				Negative	0.93 (0.44-1.95)	
		83	Creatinine clearance	≥ 60 ml/min	1.06 (0.54-2.10)	0.79
				< 60 ml/min	0.80 (0.11-5.77)	
		83	Histology	Differentiated	1.22 (0.40-3.68)	0.74
				Undifferentiated	0.97 (0.43-2.16)	
		83	Clinical T	T4a	0.91 (0.46-1.79)	

		83	Clinical N	N0	0.54 (0.08-3.89)	0.48
				N+	1.12 (0.56-2.24)	
		83	Clinical M	M0	1.09 (0.51-2.33)	0.64
				M1	0.76 (0.21-2.68)	
Dtx+S-1 vs Cis+S-1	Lee 2018[36]	153	Gender	Male	1.09 (0.60-1.98)	0.85
				Female	1.21 (0.49-3.03)	
		153	Age	< 65	1.23 (0.72-2.13)	0.29
				≥ 65	0.57 (0.15-2.13)	
		153	Performance status	0	1.29 (0.67-2.48)	0.37
				1	0.82 (0.39-1.75)	
		153	Stage	IIIA	0.84 (0.14-5.02)	0.10
				IIIB	0.58 (0.25-1.33)	
				IIIC	1.89 (0.95-3.77)	
		153	T stage	pT2-3	0.55 (0.20-1.55)	0.11
				pT4	1.45 (0.79-2.65)	
		153	N stage	pN0-2	1.92 (0.58-6.39)	0.37
				pN3	1.05 (0.61-1.83)	
Epi+Cis+5-FU+RT vs 5-FU/Lv+RT	Fuchs 2017[33]	546	Age	< Q1	1.04 (0.67-1.74)	0.29
				Q1 ≤ age < Q2	1.19 (0.43-3.27)	
				Q2 ≤ age < Q3	0.90 (0.53-1.49)	
				≥ Q3	0.57 (0.35-0.94)	
			Gender	Male	0.95 (0.73-1.28)	0.57
				Female	1.09 (0.73-1.73)	
			Race	Black	2.17 (0.93-5.06)	0.07
				White	0.95 (0.75-1.26)	
			Performance status	0	1.19 (0.85-1.69)	0.31
				1-2	0.94 (0.69-1.35)	
			T stage	T1-2	0.91 (0.63-1.36)	0.21
				T3-4	1.24 (0.91-1.65)	

			Grade	I-II	1.56 (0.92-2.60)	0.07
				III-IV	0.91 (0.70-1.20)	
			Positive nodes	1-3	0.93 (0.62-1.47)	0.90
				≥ 4	0.96 (0.73-1.36)	
			Primary tumor location	Distal gastric	0.89 (0.60-1.35)	0.65
				GEJ	1.03 (0.67-1.71)	
				Multicentric	1.36 (0.71-2.61)	
				Stomach NOS	1.31 (0.64-2.70)	
			Nodes examined	7	1.15 (0.58-2.28)	0.91
				7-14	0.97 (0.49-1.93)	
				≥ 15	1.14 (0.83-1.60)	
Surg vs 5-FU/Lv+RT	Smalley 2012[31]	559	Gender	Male	1.45 (1.16-1.82)	0.08
				Female	1.00 (0.67-1.48)	
		559	Race	Black	1.79 (1.05-3.03)	0.09
				Other	1.25 (1.00-1.54)	
		559	T stage	T1-T2	1.43 (1.00-2.04)	0.89
				T3	1.25 (0.98-1.59)	
				T4	1.04 (0.51-2.09)	
		559	N stage	N0	1.28 (0.76-2.18)	0.89
				N1-N3	1.28 (0.95-1.72)	
				N4+	1.35 (1.02-1.76)	
		551	D level	D0	1.37 (1.05-1.78)	0.53
				D1	1.35 (0.97-1.85)	
				D2+	1.07 (x-2.44)	
		553	Primary location	Proximal	1.41 (0.97-2.04)	0.82
				Other	1.32 (1.04-1.67)	
		553	Maruyama index	< 5	1.39 (0.73-2.63)	0.76
				≥ 5	1.30 (1.06-1.59)	
		432	Histology	Intestinal	1.41 (1.06-1.85)	0.08
				Diffuse	0.90 (0.63-1.30)	

		432	Gender and histology	Men, intestinal	1.39 (1.02-1.93)	0.11
				Women, intestinal	1.51 (0.71-3.10)	
				Men, diffuse	1.21 (0.76-1.96)	
				Women, diffuse	0.45 (0.23-0.88)	
Surg vs 5-FU/Lv+RT	Gordon 2013[50]	258	HER2 FISH	Non amplified	1.58 (1.17-2.14)	0.03
				Amplified	1.44 (0.44-4.75)	
			HER2 IHC	2+/3+		0.20
				0/1+		
Epi+Eto+5-FU/Lv vs Surg	De Vita 2007[8]	228	Node involvement	N+	0.84 (0.69-1.01)	
Ox+Cap (prolong) vs Ox+Cap	Feng 2015[34]	307	Stage	IIIA	0.56 (0.33-0.93)	
Doxo+5-FU+PAU vs Doxo+5-FU	Jeung 2008[19]	292	Stage	II	0.16 (0.02-1.46)	0.17
				III	0.70 (0.51-0.97)	
UFT vs Surg	Nakajima 2007[12]	190	N stage	N1	0.52 (0.26-1.05)	0.71
				N2	0.40 (0.12-1.34)	
MAGIC+CLASSIC+ARTIST+ ITACA-S (Chemo vs surgery)	Pietrantonio 2019[52]	1552	MS status	MSS	0.73 (0.61-0.86)	0.14
				MSI	1.49 (0.56-3.96)	
MAGIC+CLASSIC (Chemo vs. Surgery)		NR		MSS	0.71 (0.58-0.88)	0.03
				MSI	2.46 (0.84-7.20)	

5-FU= Fluorouracil; AJCC= American Joint Committee on Cancer; AREG= Amphiregulin; BEV= Bevacizumab; Cap= Capecitabine; Cis= Cisplatin; Doxo= Doxorubicin; Dtx= Docetaxel; DPD= Dihydropyrimidine dehydrogenase; EGFR= Epidermal growth factor; Epi= Epirubicin; Eto= Etoposide; FISH= Fluorescent in situ hybridization; GEJ= Gastroesophageal junction; HER2= Human epidermal growth factor receptor 2; HR= Hazard ratio; IGF1R= insulin-like growth factor-1; IHC= Immunohistochemistry; IRI= Irinotecan; Lv= Leucovorin; N= Number; Neo= Neoadjuvant; No.= Number; OPRT= Orotate phosphoribosyltransferase; Ox= Oxaliplatin; PAU= polyadenylic–polyuridylic acid; Peri= Perioperative; Prolong= prolonged; Ptx= Paclitaxel; RT= Radiotherapy; Surg= Surgery only; TP= Thymidine phosphorylase; TS= Thymidylate synthetase; UFT= Tegafur/uracil; UICC= International Union Against Cancer

Supplementary Table S6. Predictive factors for esophageal cancer concerning OS

Comparison	Studies	N	Factor	Categories	HR	P interaction
Neo+Epi+Cis+Cap vs Neo+Cis+5-FU	Alderson 2017[59]	629	Gender	Male	0.89 (0.76-1.05)	0.75
				Female	0.98 (0.56-1.72)	
		629	Age	< 60	1.06 (0.82-1.37)	0.05
				60-69	0.72 (0.57-0.91)	
				≥ 70	1.09 (0.76-1.58)	
		629	WHO	0	0.92 (0.76-1.12)	0.49
				1	0.82 (0.63-1.07)	
		623	T stage	T2	0.84 (0.50-1.41)	0.41
				T3	0.93 (0.79-1.10)	
				T4	0.51 (0.21-1.24)	
		624	N stage	N0	0.63 (0.45-0.90)	0.03
				N1	0.97 (0.81-1.16)	
Neo+Cis+5-FU vs Cis+5-FU	Ando 2012[62]	329	Stage	II	0.60 (0.36-0.96)	0.20
				III	0.89 (0.60-1.30)	
		330	cT stage	T1+T2	0.36 (0.17-0.80)	0.05
				T3	0.86 (0.61-1.20)	
		330	cN stage	N0	0.78 (0.46-1.32)	0.77
				N1	0.70 (0.49-1.02)	
		330	WHO	0	0.71 (0.50-1.02)	0.76
				1	0.79 (0.45-1.40)	
		330	Location	Upper	0.50 (0.18-1.47)	0.41
				Middle	0.64 (0.42-0.98)	
				Lower	0.93 (0.58-1.50)	
Neo+Eto+Cis vs Surg	Boonstra 2011[57]	169	Age	<50	1.42 (0.66-3.06)	0.18
				51-60	0.64 (0.37-1.19)	

				>60	0.63 (0.37-1.00)	
		169	Gender	Male	0.76 (0.52-1.10)	0.40
				Female	0.54 (0.27-1.10)	
		147	Weight loss	< 5%	0.79 (0.44-1.41)	0.14
				6-10%	0.91 (0.47-1.80)	
				>10%	0.40 (0.22-0.72)	
		154	Location	Upper third	1.08 (0.22-5.30)	0.14
				Middle third	0.47 (0.29-0.77)	
				Distal third	0.91 (0.56-1.50)	
		157	Tumor length	<3 cm	0.54 (0.24-1.30)	
				4-6 cm	0.71 (0.44-1.20)	0.87
				7-10	0.81 (0.47-1.40)	
				>10 cm	0.87 (0.10-7.6)	
		139	Extend of resection	R0	0.61 (0.39-0.96)	0.24
				R1	1.1 (0.56-2.16)	
				R2	1.42 (0.36-5.60)	
		139	Lymph nodes involved	pN0	0.67 (0.40-1.10)	0.51
				pN1	0.49 (0.25-0.97)	
				M1a	0.95 (0.38-2.38)	
dCRT-Ox+5-FU/Lv+RT vs	Conroy 2014[63]	270	Age	<61	0.75 (0.47-1.18)	0.18
dCRT-Cis+5-FU+RT				≥61	1.16 (0.74-1.83)	
		267	Gender	male	0.94 (0.66-1.35)	0.87
				female	0.88 (0.42-1.85)	
		266	ECOG performance score	0	0.73 (0.54-0.99)	0.59
				1-2	0.70 (0.49-1.02)	
		267	Histology	AC	0.96 (0.41-2.27)	0.94
				SCC	0.93 (0.66-1.32)	
		166	Length of tumor	<5.8 cm	1.17 (0.58-2.37)	0.74
				≥5.8 cm	1.37 (0.76-2.46)	

		243	Stage TNM	I-II	0.68 (0.36-1.28)	0.16
				III-IV	1.17 (0.78-1.76)	
dCRT-Cis+Cap+CTX+RT vs	Crosby 2017[93]	258	Age	<65	1.28 (0.78-2.10)	0.87
dCRT-Cis+Cap+RT				≥65	1.35 (0.88-2.07)	
		258	Reason for no surgery	Patients choice	1.22 (0.74-2.01)	0.18
				Local extent of disease	1.19 (0.77-1.84)	
				Comorbidity/poor PS	3.00 (1.20-7.50)	
		258	Gender	Female	0.81 (0.50-1.31)	0.01
				Male	1.87 (1.26-2.77)	
		258	WHO PS	0	1.21 (0.78-1.88)	0.58
				1	1.45 (0.90-2.34)	
		258	Histology	SCC	1.30 (0.88-1.92)	0.80
				AC/undif	1.42 (0.82-2.46)	
		258	Stage	I/II	1.37 (0.81-2.32)	0.93
				III	1.33 (0.88-2.01)	
		258	Disease length	< 4 cm	2.10 (0.99-4.46)	0.17
				4-5.9	1.02 (0.60-1.73)	
				6-7.9	1.77 (0.93-3.37)	
				≥8	0.88 (0.50-1.55)	
		257	DNLR	<2	1.23 (0.77-1.96)	0.77
				≥2	1.24 (0.67-2.28)	
Neo+5-FU+Cis+RT vs Surg	Mariette 2014[64]	195	Age	≤60	1.13 (0.71-1.76)	0.35
				>60	0.80 (0.45-1.40)	
		195	Gender	Male	1.04 (0.72-1.51)	0.36
				Female	0.63 (0.18-1.75)	
		204	Stage	I	1.13 (0.43-2.97)	0.75
				IIA/IIB	0.97 (0.65-1.40)	
		194	Histology	SCC	0.98 (0.64-1.49)	0.73
				AC	1.20 (0.60-2.40)	

		171	Lymph nodes involved	0	1.04 (0.63-1.77)	0.65
				≥1	0.88 (0.52-1.53)	
Neo+Cis+5-FU+(RT) vs	MRC 2002[58]	802	Histology	SCC	0.78 (0.59-1.05)	0.77
Neo+(RT)+Surg				AC	0.78 (0.64-0.95)	
				Undifferentiated/unknown	1.08 (0.45-2.60)	
		802	Age	<60	0.71 (0.55-0.94)	0.10
				60-69	0.97 (0.76-1.25)	
				≥70	0.64 (0.44-0.91)	
		802	Gender	Male	0.77 (0.64-0.92)	0.58
				Female	0.86 (0.61-1.20)	
		802	Site	Upper third-middle third	1.02 (0.75-1.38)	0.16
				Lower third	0.74 (0.61-0.90)	
				Cardia	0.63 (0.38-1.08)	
		753	WHO	0	0.77 (0.62-0.96)	0.74
				1-3	0.81 (0.64-1.04)	
		754	Dysphagia score	0	1.17 (0.70-1.96)	0.04
				1	0.66 (0.61-0.85)	
				2	0.76 (0.58-1.03)	
				3-4	1.30 (0.83-2.04)	
Neo+Cis+5-FU+(RT) vs	Allum 2009[97]	780	Histology	ACC	0.86 (0.71-1.05)	0.73
Neo+(RT)+Surg				SCC	0.81 (0.61-1.07)	
Neo+Ptx+Car+RT vs Surg	Shapiro 2015[61]	240	Gender	Female	0.85 (0.48-1.50)	0.43
				Male	0.66 (0.49-0.88)	
		235	Histology	SCC	0.46 (0.26-0.79)	0.11
				AC	0.75 (0.56-1.01)	
		231	cN stage	0	0.49 (0.30-0.80)	0.07
				1	0.83 (0.61-1.13)	
		240	WHO score	0	0.67 (0.51-0.90)	0.68
				1	0.79 (0.41-1.51)	

Neo+Eto+Cis+5-FU/Lv+RT vs	Stahl 2017[60]	119	Tumor location	Siewert Type I	0.71 (0.39-1.29)	0.70
Neo+Cis+5-FU/Lv				Siewert Type II	0.60 (0.32-1.14)	
		119	cT stage	T 3	0.59 (0.37-0.93)	0.25
				T4	1.67 (0.30-9.16)	
		119	Weight loss	<10%	0.71 (0.42-1.18)	0.63
				≥10%	0.56 (0.25-1.27)	
		119	Gender	Male	0.73 (0.46-1.15)	0.11
				Female	0.18 (0.03-0.95)	
		112	WHO	0	0.75 (0.43-1.31)	0.33
				1	0.47 (0.22-1.00)	
Peri+Ptx+Cis+5-FU vs	Zhao 2014[65]	346	Age	<60	0.85 (0.69-1.05)	0.89
Neo+Ptx+Cis+5-FU				60-69	0.92 (0.67-1.25)	
				≥70	0.93 (0.62-1.39)	
		346	Gender	Male	0.89 (0.75-1.07)	0.72
				Female	0.83 (0.57-1.21)	
		346	WHO	0	0.85 (0.69-1.04)	0.41
				1	0.97 (0.76-1.23)	
		346	Weight loss	≥10%	0.82 (0.46-1.45)	0.79
				<10%	0.89 (0.75-1.05)	
		346	Tumor site	Upper/middle	0.86 (0.66-1.13)	0.87
				Lower oes & GOJ	0.89 (0.73-1.08)	
		346	Tumor diameter	<8cm	0.86 (0.71-1.04)	0.54
				≥8cm	0.95 (0.73-1.24)	
Neo+Vin+Cis+RT vs Surg	Liu 2018[86]	451	Age	≤60	0.68 (0.48-0.96)	0.55
				>60	0.83 (0.48-1.42)	
		451	Gender	Male	0.80 (0.58-1.09)	0.06
				Female	0.34 (0.15-0.80)	
		451	Tumor location	Proximal third	0.51 (0.21-1.23)	0.62
				Middle third	0.70 (0.50-1.00)	

				Distal third	0.89 (0.45-1.78)	
		451	cT stage	T1-2	1.70 (0.66-4.40)	0.10
				T3	0.56 (0.38-0.82)	
				T4	0.73 (0.42-1.27)	
		451	cN stage	N0	0.56 (0.26-1.17)	0.53
				N1	0.73 (0.53-1.01)	
Peri-CTX+Neo+Dtx+Cis+RT vs	Ruhstaller 2018[88]	297	Histology	AC	0.78 (0.52-1.17)	0.57
Neo+Dtx+Cis+RT				SCC	0.64 (0.37-1.11)	
		297	Gender	Male	0.74 (0.52-1.04)	0.85
				Female	0.66 (0.22-1.98)	
		297	T stage	T2	0.88 (0.38-2.03)	0.62
				T3/4	0.70 (0.49-1.00)	
		282	Length of tumor	≤5 cm	0.83 (0.42-1.63)	0.59
				>5 cm	0.67 (0.46-0.99)	
		257	Regression grade	TRG 1	0.57 (0.29-1.11)	0.30
				TRG 2	0.94 (0.48-1.84)	
				TRG > 2	1.12 (0.65-1.94)	
Neo+Cis+5-FU vs	Dobeln 2018[87]	181	Gender	Female	1.10 (0.37-3.32)	0.87
Neo+Cis+5-FU+RT				Men	1.00 (0.67-1.50)	
		181	Histology	SCC	1.16 (0.52-2.63)	0.67
				AC	0.95 (0.62-1.46)	
		181	WHO	0	0.93 (0.62-1.39)	0.70
				1	1.26 (0.29-5.55)	
		181	Tumor location	Distal/cardia	1.13 (0.74-1.71)	0.10
				Mid/proximal	0.46 (0.17-1.22)	
		181	Age	≤60	1.64 (0.83-3.24)	0.11
				>60	0.83 (0.51-1.33)	
		181	T stage	T1-2	1.08 (0.51-2.32)	0.92

				T3	1.03 (0.65-1.63)	
		181	N stage	N0	0.81 (0.42-1.55)	0.52
				N1	1.06 (0.66-1.70)	

5-FU= Fluorouracil; AC= Adenocarcinoma; Cap= Capecitabine; Car= Carboplatin; Cis= Cisplatin; CTX= Cetuximab; dCRT= Definitive chemoradiotherapy; DNLR= Derived neutrophil to lymphocyte ratio; Epi= Epirubicin; Eto= Etoposide; GOJ= Gastro esophageal junction; HR= Hazard ratio; Peri= Perioperative; Lv= Leucovorin; N= Number; Neo= Neoadjuvant; Ox= Oxaliplatin; PS= Performance score; Ptx= Paclitaxel; RT= Radiotherapy; SCC= Squamous cell carcinoma; Surg= Surgery only; Undi= Undifferentiated; WHO= World health organization

References

1. Adjuvant treatments following curative resection for gastric cancer. The Italian Gastrointestinal Tumor Study Group. In *The British journal of surgery*, 1988; Vol. 75, pp 1100-1104.
2. Allum, W.H.; Hallissey, M.T.; Kelly, K.A. Adjuvant chemotherapy in operable gastric cancer. 5 year follow-up of first British Stomach Cancer Group trial. *Lancet* **1989**, *1*, 571-574.
3. Bajetta, E.; Buzzoni, R.; Mariani, L.; Beretta, E.; Bozzetti, F.; Bordogna, G.; Aitini, E.; Fava, S.; Schieppati, G.; Pinotti, G., et al. Adjuvant chemotherapy in gastric cancer: 5-year results of a randomised study by the Italian Trials in Medical Oncology (ITMO) Group. *Ann Oncol* **2002**, *13*, 299-307.
4. Bamias, A.; Karina, M.; Papakostas, P.; Kostopoulos, I.; Bobos, M.; Vourli, G.; Samantas, E.; Christodoulou, C.; Pentheroudakis, G.; Pectasides, D., et al. A randomized phase III study of adjuvant platinum/docetaxel chemotherapy with or without radiation therapy in patients with gastric cancer. *Cancer Chemother Pharmacol* **2010**, *65*, 1009-1021, doi:http://dx.doi.org/10.1007/s00280-010-1256-6.
5. Noh, S.H.; Park, S.R.; Yang, H.K.; Chung, H.C.; Chung, I.J.; Kim, S.W.; Kim, H.H.; Choi, J.H.; Kim, H.K.; Yu, W., et al. Adjuvant capecitabine plus oxaliplatin for gastric cancer after D2 gastrectomy (CLASSIC): 5-year follow-up of an open-label, randomised phase 3 trial. *Lancet Oncol* **2014**, *15*, 1389-1396, doi:http://dx.doi.org/10.1016/S1470-2045(14)70473-5.
6. Basi, A.; Sohrabkhani, S.; Zamani, F.; Baghai-Wadji, M.; Rabiei, N.; Razavi, S.M.; Ajdarkosh, H. Comparing Efficacy of Preoperative neo-Adjuvant Chemotherapy and Surgery versus Surgery Alone in Patients with Resectable Gastroesophageal Cancer. *Int* **2013**, *7*, 24-28.
7. Chang, H.M.; Jung, K.H.; Kim, T.Y.; Kim, W.S.; Yang, H.K.; Lee, K.U.; Choe, K.J.; Heo, D.S.; Bang, Y.J.; Kim, N.K. A phase III randomized trial of 5-fluorouracil, doxorubicin, and mitomycin C versus 5-fluorouracil and mitomycin C versus 5-fluorouracil alone in curatively resected gastric cancer. *Ann Oncol* **2002**, *13*, 1779-1785.
8. De Vita, F.; Giuliani, F.; Orditura, M.; Maiello, E.; Galizia, G.; Di Martino, N.; Montemurro, F.; Carteni, G.; Manzione, L.; Romito, S., et al. Adjuvant chemotherapy with epirubicin, leucovorin, 5-fluorouracil and etoposide regimen in resected gastric cancer patients: a randomized phase III trial by the Gruppo Oncologico Italia Meridionale (GOIM 9602 Study). *Ann Oncol* **2007**, *18*, 1354-1358.
9. Di Costanzo, F.; Gasperoni, S.; Manzione, L.; Bisagni, G.; Labianca, R.; Bravi, S.; Cortesi, E.; Carlini, P.; Bracci, R.; Tomao, S., et al. Adjuvant chemotherapy in completely resected gastric cancer: a randomized phase III trial conducted by GOIRC. *J Natl Cancer Inst* **2008**, *100*, 388-398, doi:http://dx.doi.org/10.1093/jnci/djn054.
10. Lise, M.; Nitti, D.; Marchet, A.; Sahmoud, T.; Buyse, M.; Duez, N.; Fiorentino, M.; Dos Santos, J.G.; Labianca, R.; Rougier, P., et al. Final results of a phase III clinical trial of adjuvant chemotherapy with the modified fluorouracil, doxorubicin, and mitomycin regimen in resectable gastric cancer. *J Clin Oncol* **1995**, *13*, 2757-2763.
11. Ma, J.; Yao, S.; Li, X.S.; Kang, H.R.; Yao, F.F.; Du, N. Neoadjuvant Therapy of DOF Regimen Plus Bevacizumab Can Increase Surgical Resection Ratein Locally Advanced Gastric Cancer: A Randomized, Controlled Study. *Medicine (Baltimore)* **2015**, *94*, e1489, doi:http://dx.doi.org/10.1097/MD.0000000000001489.
12. Nakajima, T.; Kinoshita, T.; Nashimoto, A.; Sairenji, M.; Yamaguchi, T.; Sakamoto, J.; Fujiya, T.; Inada, T.; Sasako, M.; Ohashi, Y., et al. Randomized controlled trial of adjuvant uracil-tegafur versus surgery alone for serosa-negative, locally advanced gastric cancer. *Br J Surg* **2007**, *94*, 1468-1476.
13. Neri, B.; Cini, G.; Andreoli, F.; Boffi, B.; Francesconi, D.; Mazzanti, R.; Medi, F.; Mercatelli, A.; Romano, S.; Siliani, L., et al. Randomized trial of adjuvant chemotherapy versus control after curative resection for gastric cancer: 5-year follow-up. *Br J Cancer* **2001**, *84*, 878-880.

14. Popiela, T.; Kulig, J.; Czupryna, A.; Szczepanik, A.M.; Zembala, M. Efficiency of adjuvant immunochemotherapy following curative resection in patients with locally advanced gastric cancer. *Gastric Cancer* **2004**, *7*, 240-245.
15. Sautner, T.; Hofbauer, F.; Depisch, D.; Schiessel, R.; Jakesz, R. Adjuvant intraperitoneal cisplatin chemotherapy does not improve long-term survival after surgery for advanced gastric cancer. *J Clin Oncol* **1994**, *12*, 970-974.
16. Nio, Y.; Koike, M.; Omori, H.; Hashimoto, K.; Itakura, M.; Yano, S.; Higami, T.; Maruyama, R. A randomized consent design trial of neoadjuvant chemotherapy with tegafur plus uracil (UFT) for gastric cancer--a single institute study. *Anticancer Res* **2004**, *24*, 1879-1887.
17. Park, S.H.; Sohn, T.S.; Lee, J.; Lim, D.H.; Hong, M.E.; Kim, K.M.; Sohn, I.; Jung, S.H.; Choi, M.G.; Lee, J.H., et al. Phase III trial to compare adjuvant chemotherapy with capecitabine and cisplatin versus concurrent chemoradiotherapy in gastric cancer: Final report of the adjuvant chemoradiotherapy in stomach tumors trial, including survival and subset analyses. In *J Clin Oncol*, 2015; Vol. 33, pp 3130-3136.
18. Bouche, O.; Ychou, M.; Burtin, P.; Bedenne, L.; Ducreux, M.; Lebreton, G.; Baulieux, J.; Nordlinger, B.; Martin, C.; Seitz, J.F., et al. Adjuvant chemotherapy with 5-fluorouracil and cisplatin compared with surgery alone for gastric cancer: 7-year results of the FFCD randomized phase III trial (8801). *Ann Oncol* **2005**, *16*, 1488-1497.
19. Jeung, H.C.; Moon, Y.W.; Rha, S.Y.; Yoo, N.C.; Roh, J.K.; Noh, S.H.; Min, J.S.; Kim, B.S.; Chung, H.C. Phase III trial of adjuvant 5-fluorouracil and adriamycin versus 5-fluorouracil, adriamycin, and polyadenylic-polyuridylic acid (poly A:U) for locally advanced gastric cancer after curative surgery: final results of 15-year follow-up. *Ann Oncol* **2008**, *19*, 520-526.
20. Krook, J.E.; O'Connell, M.J.; Wieand, H.S.; Beart, R.W., Jr.; Leigh, J.E.; Kugler, J.W.; Foley, J.F.; Pfeifle, D.M.; Twito, D.I. A prospective, randomized evaluation of intensive-course 5-fluorouracil plus doxorubicin as surgical adjuvant chemotherapy for resected gastric cancer. *Cancer* **1991**, *67*, 2454-2458.
21. Ychou, M.; Boige, V.; Pignon, J.P.; Conroy, T.; Bouche, O.; Lebreton, G.; Ducourtieux, M.; Bedenne, L.; Fabre, J.M.; Saint-Aubert, B., et al. Perioperative chemotherapy compared with surgery alone for resectable gastroesophageal adenocarcinoma: an FNCLCC and FFCD multicenter phase III trial. *J Clin Oncol* **2011**, *29*, 1715-1721, doi:http://dx.doi.org/10.1200/JCO.2010.33.0597.
22. Choi, J.S.; Lee, K.H.; Ahn, M.J.; Lee, J.S.; Lee, J.H.; Zang, D.Y.; Suh, C.W.; Kim, S.W.; Kim, W.G.; Kim, J.C., et al. A randomized trial comparing cisplatin plus 5-fluorouracil with or without levamisole in operable gastric cancer. In *The Korean journal of internal medicine*, 1997; Vol. 12, pp 155-162.
23. Kim, S.Y.; Park, H.C.; Yoon, C.; Yoon, H.J.; Choi, Y.M.; Cho, K.S. OK-432 and 5-fluorouracil, doxorubicin, and mitomycin C (FAM-P) versus FAM chemotherapy in patients with curatively resected gastric carcinoma: a randomized Phase III trial. In *Cancer*, 1998; Vol. 83, pp 2054-2059.
24. Lee, J.J.; Kim, S.Y.; Shin, I.; Cho, K.S.; Joo, H.Z.; Yoon, C. Randomized Phase III Trial of Cisplatin, Epirubicin, Leucovorin, 5-Fluorouracil (PELF) Combination versus 5-fluorouracil Alone as Adjuvant Chemotherapy in Curative Resected Stage III Gastric Cancer. In *Cancer Research and Treatment*, 2004; Vol. 36, pp 140-145.
25. Zhu, W.G.; Xua, D.F.; Pu, J.; Zong, C.D.; Li, T.; Tao, G.Z.; Ji, F.Z.; Zhou, X.L.; Han, J.H.; Wang, C.S., et al. A randomized, controlled, multicenter study comparing intensity-modulated radiotherapy plus concurrent chemotherapy with chemotherapy alone in gastric cancer patients with D2 resection. *Radiother Oncol* **2012**, *104*, 361-366, doi:http://dx.doi.org/10.1016/j.radonc.2012.08.024.
26. Kuramoto, M.; Shimada, S.; Ikeshima, S.; Matsuo, A.; Yagi, Y.; Matsuda, M.; Yonemura, Y.; Baba, H. Extensive intraoperative peritoneal lavage as a standard prophylactic strategy for peritoneal recurrence in patients with gastric carcinoma. *Ann Surg* **2009**, *250*, 242-246, doi:http://dx.doi.org/10.1097/SLA.0b013e3181b0c80e.

27. Cunningham, D.; Allum, W.H.; Stenning, S.P.; Thompson, J.N.; Van de Velde, C.J.; Nicolson, M.; Scarffe, J.H.; Lofts, F.J.; Falk, S.J.; Iveson, T.J., et al. Perioperative chemotherapy versus surgery alone for resectable gastroesophageal cancer. *N Engl J Med* **2006**, *355*, 11-20.
28. Cunningham, D.; Stenning, S.P.; Smyth, E.C.; Okines, A.F.; Allum, W.H.; Rowley, S.; Stevenson, L.; Grabsch, H.I.; Alderson, D.; Crosby, T., et al. Peri-operative chemotherapy with or without bevacizumab in operable oesophagogastric adenocarcinoma (UK Medical Research Council ST03): primary analysis results of a multicentre, open-label, randomised phase 2-3 trial. *The Lancet Oncology* **2017**, *18*, 357-370, doi:http://dx.doi.org/10.1016/S1470-2045%2817%2930043-8.
29. Bajetta, E.; Floriani, I.; Di Bartolomeo, M.; Labianca, R.; Falcone, A.; Di Costanzo, F.; Comella, G.; Amadori, D.; Pinto, C.; Carlomagno, C., et al. Randomized trial on adjuvant treatment with FOLFIRI followed by docetaxel and cisplatin versus 5-fluorouracil and folinic acid for radically resected gastric cancer. *Ann Oncol* **2014**, *25*, 1373-1378, doi:http://dx.doi.org/10.1093/annonc/mdu146.
30. Sasako, M.; Sakuramoto, S.; Katai, H.; Kinoshita, T.; Furukawa, H.; Yamaguchi, T.; Nashimoto, A.; Fujii, M.; Nakajima, T.; Ohashi, Y. Five-year outcomes of a randomized phase III trial comparing adjuvant chemotherapy with S-1 versus surgery alone in stage II or III gastric cancer. *J Clin Oncol* **2011**, *29*, 4387-4393, doi:http://dx.doi.org/10.1200/JCO.2011.36.5908.
31. Smalley, S.R.; Benedetti, J.K.; Haller, D.G.; Hundahl, S.A.; Estes, N.C.; Ajani, J.A.; Gunderson, L.L.; Goldman, B.; Martenson, J.A.; Jessup, J.M., et al. Updated analysis of SWOG-directed intergroup study 0116: a phase III trial of adjuvant radiochemotherapy versus observation after curative gastric cancer resection. *J Clin Oncol* **2012**, *30*, 2327-2333, doi:http://dx.doi.org/10.1200/JCO.2011.36.7136.
32. Yoshikawa, T.; Morita, S.; Tanabe, K.; Nishikawa, K.; Ito, Y.; Matsui, T.; Fujitani, K.; Kimura, Y.; Fujita, J.; Aoyama, T., et al. Survival results of a randomised two-by-two factorial phase II trial comparing neoadjuvant chemotherapy with two and four courses of S-1 plus cisplatin (SC) and paclitaxel plus cisplatin (PC) followed by D2 gastrectomy for resectable advanced gastric cancer. *Eur J Cancer* **2016**, *62*, 103-111, doi:http://dx.doi.org/10.1016/j.ejca.2016.04.012.
33. Fuchs, C.S.; Niedzwiecki, D.; Mamon, H.J.; Tepper, J.E.; Ye, X.; Swanson, R.S.; Enzinger, P.C.; Haller, D.G.; Dragovich, T.; Alberts, S.R., et al. Adjuvant Chemoradiotherapy With Epirubicin, Cisplatin, and Fluorouracil Compared With Adjuvant Chemoradiotherapy With Fluorouracil and Leucovorin After Curative Resection of Gastric Cancer: Results From CALGB 80101 (Alliance). *J Clin Oncol* **2017**, *35*, 3671-3677, doi:10.1200/JCO.2017.74.2130.
34. Feng, W.M.; Tang, C.W.; Guo, H.H.; Bao, Y.; Fei, M.Y. Prolonged adjuvant capecitabine chemotherapy improved survival of stage IIIA gastric cancer after D2 gastrectomy. *Biomed Pharmacother* **2015**, *72*, 140-143, doi:http://dx.doi.org/10.1016/j.biopha.2015.03.003.
35. Hallissey, M.T.; Dunn, J.A.; Ward, L.C.; Allum, W.H. The second British Stomach Cancer Group trial of adjuvant radiotherapy or chemotherapy in resectable gastric cancer: five-year follow-up. *Lancet* **1994**, *343*, 1309-1312.
36. Lee, C.K.; Jung, M.; Kim, H.S.; Jung, I.; Shin, D.B.; Kang, S.Y.; Zang, D.Y.; Kim, K.H.; Lee, M.H.; Kim, B.S., et al. S-1 Based Doublet as an Adjuvant Chemotherapy for Curatively Resected Stage III Gastric Cancer: Results from the Randomized Phase III POST Trial. *Cancer Res Treat* **2018**, 10.4143/crt.2018.028, doi:10.4143/crt.2018.028.
37. Cats, A.; Jansen, E.P.M.; van Grieken, N.C.T.; Sikorska, K.; Lind, P.; Nordsmark, M.; Meershoek-Klein Kranenbarg, E.; Boot, H.; Trip, A.K.; Swellengrebel, H.A.M., et al. Chemotherapy versus chemoradiotherapy after surgery and preoperative chemotherapy for resectable gastric cancer (CRITICS): an international, open-label, randomised phase 3 trial. *Lancet Oncol* **2018**, *19*, 616-628, doi:10.1016/S1470-2045(18)30132-3.
38. Nakajima, T.; Nashimoto, A.; Kitamura, M.; Kito, T.; Iwanaga, T.; Okabayashi, K.; Goto, M. Adjuvant mitomycin and fluorouracil followed by oral uracil plus tegafur in serosa-negative gastric cancer: a randomised trial. Gastric Cancer Surgical Study Group. *Lancet* **1999**, *354*, 273-277.

39. Grau, J.J.; Estape, J.; Fuster, J.; Filella, X.; Visa, J.; Teres, J.; Soler, G.; Albiol, S.; Garcia-Valdecasas, J.C.; Grande, L., et al. Randomized trial of adjuvant chemotherapy with mitomycin plus ftorafur versus mitomycin alone in resected locally advanced gastric cancer. *J Clin Oncol* **1998**, *16*, 1036-1039.
40. Yu, C.C.; Levison, D.A.; Dunn, J.A.; Ward, L.C.; Demonakou, M.; Allum, W.H.; Hallisey, M.T. Pathological prognostic factors in the second British Stomach Cancer Group trial of adjuvant therapy in resectable gastric cancer. In *Br J Cancer*, 1995; Vol. 71, pp 1106-1110.
41. Di Bartolomeo, M.; Pietrantonio, F.; Pellegrinelli, A.; Martinetti, A.; Mariani, L.; Daidone, M.G.; Bajetta, E.; Pelosi, G.; de Braud, F.; Floriani, I., et al. Osteopontin, E-cadherin, and beta-catenin expression as prognostic biomarkers in patients with radically resected gastric cancer. *Gastric Cancer* **2016**, *19*, 412-420, doi:http://dx.doi.org/10.1007/s10120-015-0495-y.
42. Hundahl, S.A.; Macdonald, J.S.; Benedetti, J.; Fitzsimmons, T.; Southwest Oncology, G.; the Gastric, I. Surgical treatment variation in a prospective, randomized trial of chemoradiotherapy in gastric cancer: the effect of undertreatment. *Ann Surg Oncol* **2002**, *9*, 278-286.
43. Yamamura, Y.; Nakajima, T.; Ohta, K.; Nashimoto, A.; Arai, K.; Hiratsuka, M.; Sasako, M.; Kodera, Y.; Goto, M. Determining prognostic factors for gastric cancer using the regression tree method. *Gastric Cancer* **2002**, *5*, 201-207.
44. Kim, Y.; Park, S.H.; Kim, K.M.; Choi, M.G.; Lee, J.H.; Sohn, T.S.; Bae, J.M.; Kim, S.; Lee, S.J.; Kim, S.T., et al. The influence of metastatic lymph node ratio on the treatment outcomes in the Adjuvant Chemoradiotherapy in Stomach Tumors (ARTIST) trial: A phase III trial. *Journal of Gastric Cancer* **2016**, *16*, 105-110, doi:http://dx.doi.org/10.5230/jgc.2016.16.2.105.
45. Ichikawa, W.; Terashima, M.; Ochiai, A.; Kitada, K.; Kurahashi, I.; Sakuramoto, S.; Katai, H.; Sano, T.; Imamura, H.; Sasako, M. Impact of insulin-like growth factor-1 receptor and amphiregulin expression on survival in patients with stage II/III gastric cancer enrolled in the Adjuvant Chemotherapy Trial of S-1 for Gastric Cancer. *Gastric Cancer* **2017**, *20*, 263-273, doi:http://dx.doi.org/10.1007/s10120-016-0600-x.
46. Terashima, M.; Kitada, K.; Ochiai, A.; Ichikawa, W.; Kurahashi, I.; Sakuramoto, S.; Katai, H.; Sano, T.; Imamura, H.; Sasako, M., et al. Impact of expression of human epidermal growth factor receptors EGFR and ERBB2 on survival in stage II/III gastric cancer. *Clin Cancer Res* **2012**, *18*, 5992-6000, doi:10.1158/1078-0432.CCR-12-1318.
47. Okines, A.F.; Thompson, L.C.; Cunningham, D.; Wotherspoon, A.; Reis-Filho, J.S.; Langley, R.E.; Waddell, T.S.; Noor, D.; Eltahir, Z.; Wong, R., et al. Effect of HER2 on prognosis and benefit from peri-operative chemotherapy in early oesophago-gastric adenocarcinoma in the MAGIC trial. *Ann Oncol* **2013**, *24*, 1253-1261, doi:10.1093/annonc/mds622.
48. Sasako, M.; Terashima, M.; Ichikawa, W.; Ochiai, A.; Kitada, K.; Kurahashi, I.; Sakuramoto, S.; Katai, H.; Sano, T.; Imamura, H. Impact of the expression of thymidylate synthase and dihydropyrimidine dehydrogenase genes on survival in stage II/III gastric cancer. *Gastric Cancer* **2015**, *18*, 538-548, doi:10.1007/s10120-014-0413-8.
49. Smyth, E.C.; Fassan, M.; Cunningham, D.; Allum, W.H.; Okines, A.F.; Lampis, A.; Hahne, J.C.; Rugge, M.; Peckitt, C.; Nankivell, M., et al. Effect of Pathologic Tumor Response and Nodal Status on Survival in the Medical Research Council Adjuvant Gastric Infusional Chemotherapy Trial. *J Clin Oncol* **2016**, *34*, 2721-2727, doi:10.1200/JCO.2015.65.7692.
50. Gordon, M.A.; Gundacker, H.M.; Benedetti, J.; Macdonald, J.S.; Baranda, J.C.; Levin, W.J.; Blanke, C.D.; Elatre, W.; Weng, P.; Zhou, J.Y., et al. Assessment of HER2 gene amplification in adenocarcinomas of the stomach or gastroesophageal junction in the INT-0116/SWOG9008 clinical trial. *Ann Oncol* **2013**, *24*, 1754-1761, doi:10.1093/annonc/mdt106.
51. Grau, J.J.; Domingo-Domenech, J.; Morente, V.; Pera, M.; Garcia-Valdecasas, J.C.; Fuster, J.; Bombi, A.; Mellado, B.; Albanell, J.; Gascon, P. Low thymidylate synthase expression in the primary tumor predicts favorable clinical outcome in resected gastric cancer patients treated with adjuvant tegafur. *Oncology* **2004**, *66*, 226-233.

52. Pietrantonio, F.; Raimondi, A.; Choi, Y.Y.; Kang, W.; Langley, R.E.; Kim, Y.W.; Kim, K.-M.; Nankivell, M.G.; Perrone, F.; Kook, M.-C., et al. MSI-GC-01: Individual patient data (IPD) meta-analysis of microsatellite instability (MSI) and gastric cancer (GC) from four randomized clinical trials (RCTs). **2019**, *37*, 66-66, doi:10.1200/JCO.2019.37.4_suppl.66.
53. Claassen, Y.H.M.; van Amelsfoort, R.M.; Hartgrink, H.H.; Dikken, J.L.; de Steur, W.O.; van Sandick, J.W.; van Grieken, N.C.T.; Cats, A.; Boot, H.; Trip, A.K., et al. Effect of Hospital Volume With Respect to Performing Gastric Cancer Resection on Recurrence and Survival: Results from the CRITICS Trial. *Ann Surg* **2018**, *07*, 10, doi:http://dx.doi.org/10.1097/sla.0000000000002940.
54. Park, H.S.; Kim, H.S.; Beom, S.H.; Rha, S.Y.; Chung, H.C.; Kim, J.H.; Chun, Y.J.; Lee, S.W.; Choe, E.A.; Heo, S.J., et al. Marked Loss of Muscle, Visceral Fat, or Subcutaneous Fat After Gastrectomy Predicts Poor Survival in Advanced Gastric Cancer: Single-Center Study from the CLASSIC Trial. *Ann Surg Oncol* **2018**, *25*, 3222-3230, doi:http://dx.doi.org/10.1245/s10434-018-6624-1.
55. Kim, M.H.; Zhang, X.; Jung, M.; Jung, I.; Park, H.S.; Beom, S.H.; Kim, H.S.; Rha, S.Y.; Kim, H.; Choi, Y.Y., et al. Immunohistochemistry Biomarkers Predict Survival in Stage II/III Gastric Cancer Patients: From a Prospective Clinical Trial. *Cancer Res* **2018**, *27*, 27, doi:http://dx.doi.org/10.4143/crt.2018.331.
56. Popiela, T.; Kulig, J.; Czupryna, A.; Szczepanik, A.M.; Zembala, M. Efficiency of adjuvant immunochemotherapy following curative resection in patients with locally advanced gastric cancer. In *Gastric cancer : official journal of the International Gastric Cancer Association and the Japanese Gastric Cancer Association*, 2004; Vol. 7, pp 240-245.
57. Boonstra, J.J.; Kok, T.C.; Wijnhoven, B.P.; van Heijl, M.; van Berge Henegouwen, M.I.; Ten Kate, F.J.; Siersema, P.D.; Dinjens, W.N.; van Lanschot, J.J.; Tilanus, H.W., et al. Chemotherapy followed by surgery versus surgery alone in patients with resectable oesophageal squamous cell carcinoma: long-term results of a randomized controlled trial. *BMC Cancer* **2011**, *11*, 181, doi:http://dx.doi.org/10.1186/1471-2407-11-181.
58. Medical Research Council Oesophageal Cancer Working, G. Surgical resection with or without preoperative chemotherapy in oesophageal cancer: a randomised controlled trial. *Lancet* **2002**, *359*, 1727-1733.
59. Alderson, D.; Cunningham, D.; Nankivell, M.; Blazeby, J.M.; Griffin, S.M.; Crellin, A.; Grabsch, H.I.; Langer, R.; Pritchard, S.; Okines, A., et al. Neoadjuvant cisplatin and fluorouracil versus epirubicin, cisplatin, and capecitabine followed by resection in patients with oesophageal adenocarcinoma (UK MRC OE05): an open-label, randomised phase 3 trial. *Lancet Oncol* **2017**, *18*, 1249-1260, doi:10.1016/S1470-2045(17)30447-3.
60. Stahl, M.; Walz, M.K.; Riera-Knorrenschild, J.; Stuschke, M.; Sandermann, A.; Bitzer, M.; Wilke, H.; Budach, W. Preoperative chemotherapy versus chemoradiotherapy in locally advanced adenocarcinomas of the oesophagogastric junction (POET): Long-term results of a controlled randomised trial. *Eur J Cancer* **2017**, *81*, 183-190, doi:http://dx.doi.org/10.1016/j.ejca.2017.04.027.
61. Shapiro, J.; van Lanschot, J.J.; Hulshof, M.C.; van Hagen, P.; van Berge Henegouwen, M.I.; Wijnhoven, B.P.; van Laarhoven, H.W.; Nieuwenhuijzen, G.A.; Hospers, G.A.; Bonenkamp, J.J., et al. Neoadjuvant chemoradiotherapy plus surgery versus surgery alone for oesophageal or junctional cancer (CROSS): long-term results of a randomised controlled trial. *Lancet Oncol* **2015**, *16*, 1090-1098, doi:http://dx.doi.org/10.1016/S1470-2045(15)00040-6.
62. Ando, N.; Kato, H.; Igaki, H.; Shinoda, M.; Ozawa, S.; Shimizu, H.; Nakamura, T.; Yabusaki, H.; Aoyama, N.; Kurita, A., et al. A randomized trial comparing postoperative adjuvant chemotherapy with cisplatin and 5-fluorouracil versus preoperative chemotherapy for localized advanced squamous cell carcinoma of the thoracic esophagus (JCOG9907). *Ann Surg Oncol* **2012**, *19*, 68-74, doi:http://dx.doi.org/10.1245/s10434-011-2049-9.
63. Conroy, T.; Galais, M.P.; Raoul, J.L.; Bouche, O.; Gourgou-Bourgade, S.; Douillard, J.Y.; Etienne, P.L.; Boige, V.; Martel-Lafay, I.; Michel, P., et al. Definitive chemoradiotherapy with

FOLFOX versus fluorouracil and cisplatin in patients with oesophageal cancer (PRODIGE5/ACCORD17): Final results of a randomised, phase 2/3 trial. *The Lancet Oncology* **2014**, *15*, 305-314, doi:http://dx.doi.org/10.1016/S1470-2045%2814%2970028-2.
64. Mariette, C.; Dahan, L.; Mornex, F.; Maillard, E.; Thomas, P.A.; Meunier, B.; Boige, V.; Pezet, D.; Robb, W.B.; Le Brun-Ly, V., et al. Surgery alone versus chemoradiotherapy followed by surgery for stage I and II esophageal cancer: final analysis of randomized controlled phase III trial FFCD 9901. *J Clin Oncol* **2014**, *32*, 2416-2422, doi:http://dx.doi.org/10.1200/JCO.2013.53.6532.
65. Zhao, Y.; Sui, X. Perioperative versus preoperative chemotherapy with surgery in patients with resectable squamous-cell carcinoma of esophagus: A phase III randomized trial. In *J Clin Oncol*, 2014; Vol. 32.
66. al-Sarraf, M.; Martz, K.; Herskovic, A.; Leichman, L.; Brindle, J.S.; Vaitkevicius, V.K.; Cooper, J.; Byhardt, R.; Davis, L.; Emami, B. Progress report of combined chemoradiotherapy versus radiotherapy alone in patients with esophageal cancer: an intergroup study.[Erratum appears in J Clin Oncol 1997 Feb;15(2):866]. *J Clin Oncol* **1997**, *15*, 277-284.
67. Arnott, S.J.; Duncan, W.; Kerr, G.R.; Walbaum, P.R.; Cameron, E.; Jack, W.J.; Mackillop, W.J. Low dose preoperative radiotherapy for carcinoma of the oesophagus: results of a randomized clinical trial. *Radiother Oncol* **1992**, *24*, 108-113.
68. Baba, M.; Natsugoe, S.; Shimada, M.; Nakano, S.; Kusano, C.; Fukumoto, T.; Aikou, T.; Akazawa, K. Prospective evaluation of preoperative chemotherapy in resectable squamous cell carcinoma of the thoracic esophagus. *Dis Esophagus* **2000**, *13*, 136-141.
69. Badwe, R.A.; Sharma, V.; Bhansali, M.S.; Dinshaw, K.A.; Patil, P.K.; Dalvi, N.; Rayabhattanavar, S.G.; Desai, P.B. The quality of swallowing for patients with operable esophageal carcinoma: A randomized trial comparing surgery with radiotherapy. *Cancer* **1999**, *85*, 763-768, doi:http://dx.doi.org/10.1002/%28SICI%291097-0142%2819990215%2985:4%3C763::AID-CNCR2%3E3.0.CO;2-R.
70. Bass, G.A.; Furlong, H.; O'Sullivan, K.E.; Hennessy, T.P.; Walsh, T.N. Chemoradiotherapy, with adjuvant surgery for local control, confers a durable survival advantage in adenocarcinoma and squamous cell carcinoma of the oesophagus. *Eur J Cancer* **2014**, *50*, 1065-1075, doi:http://dx.doi.org/10.1016/j.ejca.2013.12.022.
71. Burmeister, B.H.; Smithers, B.M.; Gebski, V.; Fitzgerald, L.; Simes, R.J.; Devitt, P.; Ackland, S.; Gotley, D.C.; Joseph, D.; Millar, J., et al. Surgery alone versus chemoradiotherapy followed by surgery for resectable cancer of the oesophagus: a randomised controlled phase III trial. *Lancet Oncol* **2005**, *6*, 659-668.
72. Iizuka, T.; Ide, H.; Kakegawa, T.; Sasaki, K.; Takagi, I.; Ando, N.; Mori, S.; Arimori, M.; Tsugane, S. Preoperative radioactive therapy for esophageal carcinoma. Randomized evaluation trial in eight institutions. In *Chest*, 1988; Vol. 93, pp 1054-1058.
73. Ogoshi, K.; Satou, H.; Isono, K.; Mitomi, T.; Endoh, M.; Sugita, M. Immunotherapy for esophageal cancer. A randomized trial in combination with radiotherapy and radiochemotherapy. Cooperative Study Group for Esophageal Cancer in Japan. In *American journal of clinical oncology*, 1995; Vol. 18, pp 216-222.
74. Shi, X.H.; He, S.Q.; Yao, W.Q.; Wang, Y.; Guo, X.M.; Wu, G.D.; Zhu, L.X.; Liu, T.F. Comparison between continuous accelerated hyperfractionated and late-course accelerated hyperfractionated radiotherapy for esophageal carcinoma. *International Journal of Radiation Oncology Biology Physics* **2002**, *54*, 131-136, doi:http://dx.doi.org/10.1016/S0360-3016%2802%2902892-4.
75. Stahl, M.; Stuschke, M.; Lehmann, N.; Meyer, H.J.; Walz, M.K.; Seeber, S.; Klump, B.; Budach, W.; Teichmann, R.; Schmitt, M., et al. Chemoradiation with and without surgery in patients with locally advanced squamous cell carcinoma of the esophagus.[Erratum appears in J Clin Oncol. 2006 Jan 20;24(3):531]. *J Clin Oncol* **2005**, *23*, 2310-2317.

76. Urba, S.G.; Orringer, M.B.; Turrisi, A.; Iannettoni, M.; Forastiere, A.; Strawderman, M. Randomized trial of preoperative chemoradiation versus surgery alone in patients with locoregional esophageal carcinoma. *J Clin Oncol* **2001**, *19*, 305-313.
77. Teoh, A.Y.B.; Chiu, P.W.Y.; Yeung, W.K.; Liu, S.Y.W.; Wong, S.K.H.; Ng, E.K.W. Long-term survival outcomes after definitive chemoradiation versus surgery in patients with resectable squamous carcinoma of the esophagus: Results from a randomized controlled trial. *Ann Oncol* **2013**, *24*, 165-171, doi:http://dx.doi.org/10.1093/annonc/mds206.
78. Kumar, S.; Dimri, K.; Khurana, R.; Rastogi, N.; Das, K.J.M.; Lal, P. A randomised trial of radiotherapy compared with cisplatin chemo-radiotherapy in patients with unresectable squamous cell cancer of the esophagus>. *Radiotherapy and Oncology* **2007**, *83*, 139-147, doi:http://dx.doi.org/10.1016/j.radonc.2007.03.013.
79. Lee, J.L.; Park, S.I.; Kim, S.B.; Jung, H.Y.; Lee, G.H.; Kim, J.H.; Song, H.Y.; Cho, K.J.; Kim, W.K.; Lee, J.S., et al. A single institutional phase III trial of preoperative chemotherapy with hyperfractionation radiotherapy plus surgery versus surgery alone for resectable esophageal squamous cell carcinoma. *Ann Oncol* **2004**, *15*, 947-954.
80. Bosset, J.F.; Gignoux, M.; Triboulet, J.P.; Tiret, E.; Mantion, G.; Elias, D.; Lozach, P.; Ollier, J.C.; Pavy, J.J.; Mercier, M., et al. Chemoradiotherapy followed by surgery compared with surgery alone in squamous-cell cancer of the esophagus. *N Engl J Med* **1997**, *337*, 161-167.
81. Ma, D.Y.; Tan, B.X.; Liu, M.; Li, X.F.; Zhou, Y.Q.; Lu, Y. Concurrent three-dimensional conformal radiotherapy and chemotherapy for postoperative recurrence of mediastinal lymph node metastases in patients with esophageal squamous cell carcinoma: A phase 2 single-institution study. *Radiation Oncology* **2014**, *9 (1) (no pagination)*, doi:http://dx.doi.org/10.1186/1748-717X-9-28.
82. Kelsen, D.P.; Winter, K.A.; Gunderson, L.L.; Mortimer, J.; Estes, N.C.; Haller, D.G.; Ajani, J.A.; Kocha, W.; Minsky, B.D.; Roth, J.A., et al. Long-term results of RTOG trial 8911 (USA Intergroup 113): a random assignment trial comparison of chemotherapy followed by surgery compared with surgery alone for esophageal cancer. *J Clin Oncol* **2007**, *25*, 3719-3725.
83. Wang, S.; Wang, Z.; Yang, Z.; Liu, Y.; Liu, X.; Shang, B.; Jiang, W.P. Postoperative Radiotherapy Improves Survival in Stage pT2N0M0 Esophageal Squamous Cell Carcinoma with High Risk of Poor Prognosis. *Ann Surg Oncol* **2016**, *23*, 265-272, doi:http://dx.doi.org/10.1245/s10434-015-4622-0.
84. Crosby, T.; Hurt, C.N.; Falk, S.; Gollins, S.; Mukherjee, S.; Staffurth, J.; Ray, R.; Bashir, N.; Bridgewater, J.A.; Geh, J.I., et al. Chemoradiotherapy with or without cetuximab in patients with oesophageal cancer (SCOPE1): a multicentre, phase 2/3 randomised trial. In *The Lancet. Oncology*, 2013; Vol. 14, pp 627-637.
85. Xiao, Z.F.; Yang, Z.Y.; Liang, J.; Miao, Y.J.; Wang, M.; Yin, W.B.; Gu, X.Z.; Zhang, D.C.; Zhang, R.G.; Wang, L.J. Value of radiotherapy after radical surgery for esophageal carcinoma: a report of 495 patients. *Ann Thorac Surg* **2003**, *75*, 331-336.
86. Liu, H.; Chen, Y.; Zhu, C.; Fang, W.; Yu, Z.; Mao, W.; Xiang, J.; Han, Y.; Chen, Z.; Wang, J., et al. Neoadjuvant chemoradiotherapy followed by surgery versus surgery alone for locally advanced squamous cell carcinoma of the esophagus (NEOCRTEC5010): A phase III multicenter, randomized, open-label clinical trial. *J Clin Oncol* **2018**, *36*, 2796-2803, doi:http://dx.doi.org/10.1200/jco.2018.79.1483.
87. von Dobeln, G.A.; Klevebro, F.; Jacobsen, A.B.; Johannessen, H.O.; Nielsen, N.H.; Johnsen, G.; Hatlevoll, I.; Glenjen, N.I.; Friesland, S.; Lundell, L., et al. Neoadjuvant chemotherapy versus neoadjuvant chemoradiotherapy for cancer of the esophagus or gastroesophageal junction: long-term results of a randomized clinical trial. *Dis Esophagus* **2019**, *32*, 01, doi:http://dx.doi.org/10.1093/dote/doy078.
88. Ruhstaller, T.; Thuss-Patience, P.; Hayoz, S.; Schacher, S.; Knorrenschild, J.R.; Schnider, A.; Plasswilm, L.; Budach, W.; Eisterer, W.; Hawle, H., et al. Neoadjuvant chemotherapy followed by chemoradiation and surgery with and without cetuximab in patients with resectable

esophageal cancer: a randomized, open-label, phase III trial (SAKK 75/08). *Ann Oncol* **2018**, *29*, 1386-1393, doi:http://dx.doi.org/10.1093/annonc/mdy105.
89. Suntharalingam, M.; Winter, K.; Ilson, D.; Dicker, A.P.; Kachnic, L.; Konski, A.; Chakravarthy, A.B.; Anker, C.J.; Thakrar, H.; Horiba, N., et al. Effect of the Addition of Cetuximab to Paclitaxel, Cisplatin, and Radiation Therapy for Patients With Esophageal Cancer: the NRG Oncology RTOG 0436 Phase 3 Randomized Clinical Trial. *JAMA oncology* **2017**, *3*, 1520-1528, doi:http://dx.doi.org/10.1001/jamaoncol.2017.1598.
90. Kataoka, K.; Takeuchi, H.; Mizusawa, J.; Igaki, H.; Ozawa, S.; Abe, T.; Nakamura, K.; Kato, K.; Ando, N.; Kitagawa, Y. Prognostic impact of postoperative morbidity after esophagectomy for esophageal cancer: Exploratory analysis of JCOG9907. *Ann Surg* **2017**, *265*, 1152-1157, doi:http://dx.doi.org/10.1097/SLA.0000000000001828.
91. Zhang, W.; Zhu, H.; Liu, X.; Wang, Q.; Zhang, X.; He, J.; Sun, K.; Liu, X.; Zhou, Z.; Xu, N., et al. Epidermal growth factor receptor is a prognosis predictor in patients with esophageal squamous cell carcinoma. *Ann Thorac Surg* **2014**, *98*, 513-519, doi:http://dx.doi.org/10.1016/j.athoracsur.2014.03.015.
92. Robb, W.B.; Dahan, L.; Mornex, F.; Maillard, E.; Thomas, P.A.; Meunier, B.; Boige, V.; Pezet, D.; Brun-Ly, V.; Bosset, J.F., et al. Impact of neoadjuvant chemoradiation on lymph node status in esophageal cancer: post hoc analysis of a randomized controlled trial. In *Ann Surg*, 2015; Vol. 261, pp 902-908.
93. Crosby, T.; Hurt, C.N.; Falk, S.; Gollins, S.; Staffurth, J.; Ray, R.; Bridgewater, J.A.; Geh, J.I.; Cunningham, D.; Blazeby, J., et al. Long-term results and recurrence patterns from SCOPE-1: A phase II/III randomised trial of definitive chemoradiotherapy +/-cetuximab in oesophageal cancer. *Br J Cancer* **2017**, *116*, 709-716, doi:http://dx.doi.org/10.1038/bjc.2017.21.
94. Cox, S.; Hurt, C.; Grenader, T.; Mukherjee, S.; Bridgewater, J.; Crosby, T. The prognostic value of derived neutrophil to lymphocyte ratio in oesophageal cancer treated with definitive chemoradiotherapy. *Radiother Oncol* **2017**, *125*, 154-159, doi:10.1016/j.radonc.2017.08.023.
95. Cox, S.; Powell, C.; Carter, B.; Hurt, C.; Mukherjee, S.; Crosby, T. Role of nutritional status and intervention in oesophageal cancer treated with definitive chemoradiotherapy: outcomes from SCOPE1. In *Br J Cancer*, 2016; Vol. 115, pp 172-177.
96. Bascoul-Mollevi, C.; Gourgou, S.; Galais, M.P.; Raoul, J.L.; Bouche, O.; Douillard, J.Y.; Adenis, A.; Etienne, P.L.; Juzyna, B.; Bedenne, L., et al. Health-related quality of life results from the PRODIGE 5/ACCORD 17 randomised trial of FOLFOX versus fluorouracil-cisplatin regimen in oesophageal cancer. *Eur J Cancer* **2017**, *84*, 239-249, doi:10.1016/j.ejca.2017.07.038.
97. Allum, W.H.; Stenning, S.P.; Bancewicz, J.; Clark, P.I.; Langley, R.E. Long-term results of a randomized trial of surgery with or without preoperative chemotherapy in esophageal cancer. *J Clin Oncol* **2009**, *27*, 5062-5067, doi:http://dx.doi.org/10.1200/JCO.2009.22.2083.
98. Davarzani, N.; Hutchins, G.G.A.; West, N.P.; Hewitt, L.C.; Nankivell, M.; Cunningham, D.; Allum, W.H.; Smyth, E.; Valeri, N.; Langley, R.E., et al. Prognostic value of pathological lymph node status and primary tumour regression grading following neoadjuvant chemotherapy - results from the MRC OE02 oesophageal cancer trial. *Histopathology* **2018**, *72*, 1180-1188, doi:http://dx.doi.org/10.1111/his.13491.
99. Lee, S.; Park, S.H.; Lee, J.; Kang, W.K. Prognostic value of the metastatic lymph node (N) ratio in the adjuvant chemoradiotherapy in stomach tumors (ARTIST) phase III trial. *Journal of Clinical Oncology. Conference* **2016**, *34*.
100. Kataoka, K.; Takeuchi, H.; Mizusawa, J.; Igaki, H.; Ozawa, S.; Abe, T.; Nakamura, K.; Kato, K.; Ando, N.; Kitagawa, Y. Prognostic Impact of Postoperative Morbidity After Esophagectomy for Esophageal Cancer: Exploratory Analysis of JCOG9907. *Ann Surg* **2016**, *8*, 8, doi:http://dx.doi.org/10.1097/SLA.0000000000001828.
101. Arnott, S.J.; Duncan, W.; Gignoux, M.; Girling, D.J.; Hansen, H.S.; Launois, B.; Nygaard, K.; Parmar, M.K.; Roussel, A.; Spiliopoulos, G., et al. Preoperative radiotherapy in esophageal carcinoma: a meta-analysis using individual patient data (Oesophageal Cancer Collaborative Group). *Int J Radiat Oncol Biol Phys* **1998**, *41*, 579-583.

102. Cox, S.; Powell, C.; Carter, B.; Hurt, C.; Mukherjee, S.; Crosby, T.D. Role of nutritional status and intervention in oesophageal cancer treated with definitive chemoradiotherapy: outcomes from SCOPE1. *Br J Cancer* **2016**, *115*, 172-177, doi:10.1038/bjc.2016.129.
